# Diversity and Spatiotemporal Activity Patterns of Medium and Large Mammals in the Niokolo‐Koba National Park, Senegal

**DOI:** 10.1002/ece3.74005

**Published:** 2026-07-10

**Authors:** Lisa Ohrndorf, Augustin Brouillet, Annika M. Zuleger, Ndiouga Diakhaté, Djibril Coly, Chérif Younousse Kéba Camara, Amadou Bamba Diedhiou, Irene Gutiérrez Díez, Julia Fischer, Dietmar Zinner

**Affiliations:** ^1^ Department for Primate Cognition, Johann‐Friedrich‐Blumenbach Institute Georg‐August‐Universität Göttingen Göttingen Germany; ^2^ German Primate Center—Leibniz Institute for Primate Research Cognitive Ethology Laboratory Göttingen Germany; ^3^ Institute of Biology Martin Luther University Halle‐Wittenberg Halle (Saale) Germany; ^4^ German Centre for Integrative Biodiversity Research (iDiv) Halle‐Jena‐Leipzig Leipzig Germany; ^5^ Direction Des Parcs Nationaux (DPN) Dakar Senegal; ^6^ Centro de Cría del Lince Ibérico Zarza de Granadilla, Tragsatec Cáceres Spain

**Keywords:** biodiversity, camera‐trap, conservation, ecology, habitat use, West Africa

## Abstract

West African savannahs provide habitats to diverse species assemblages, yet remain understudied compared to their East and Southern African counterparts. The Niokolo‐Koba National Park in southeastern Senegal constitutes one of the largest remaining protected areas in West Africa and supports a mosaic of savannah and forest habitats with a diverse assemblage of medium‐ and large‐sized mammals. Here, we analysed camera‐trap data originally collected to monitor predator presence in the northwestern sector of the National Park. We deployed 37 cameras across 37 km^2^ from February 2022 to March 2023, resulting in 13,080 camera‐trap days. We assessed diversity indices and spatiotemporal activity patterns of large and medium‐sized mammals across habitat types. Evenness values—the degree to which species abundances are distributed uniformly within a community—were higher in the savannah than in forest habitats, although overall species richness was comparable. Estimated diel activity mostly corresponded with established species‐specific behavioural patterns. Our analyses revealed differential use of certain habitat types throughout the day, likely driven by spatially segregated sleeping and foraging sites. Our results provide a reference for future studies and monitoring efforts and highlight the value of the forest‐savannah mosaic for the local species assemblage within the larger ecosystem of Niokolo‐Koba National Park.

## Graphical abstract

The Niokolo‐Koba National Park in southeastern Senegal, a mosaic of savannah and forest habitats, supports a diverse assemblage of medium‐ and large‐sized mammals. Our camera‐trap survey revealed comparable species richness across habitats, higher evenness in savannahs and during the wet season, and generally consistent sighting rates across habitats and seasons, with diel activity largely matching species‐specific behavioural patterns. Our findings highlight the importance of the forest‐savannah mosaic and provide a reference for future monitoring and conservation efforts.

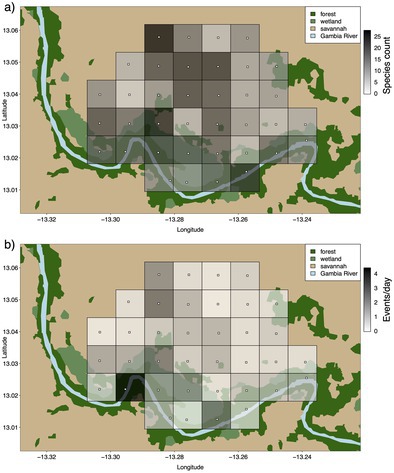

## 1 Introduction

Savannahs and savannah‐forest mosaics are among the world's largest biomes, covering up to 30% of the overall land surface (Shorrocks 2007; Bond 2019). Most of these savannahs are located in Africa, providing habitats for a wide range of medium and large mammal species. However, savannah ecosystems, especially in eastern and western Africa, are under increasing pressure, resulting in a sharp decline in large‐mammal populations over recent decades and contributing to the global decline in biodiversity (Craigie et al. 2010). Habitat loss due to overexploitation and fragmentation is among the main drivers of this decline and disproportionately affects wide‐ranging and large‐bodied species (Scholte et al. 2025). Savannahs are thus central to conservation planning across Africa. At the same time, savannah ecosystems are often overlooked or relegated to a secondary role in conservation and restoration planning (Buisson et al. 2019; Dudley et al. 2020). While savannah ecosystems in East and Southern Africa have been extensively studied within protected areas, similar research in West Africa remains limited (Bauer et al. 2021). This lack of data is concerning, given the continued decline of mammal populations in the region and the growing need to identify and protect remaining areas of ecological importance (Craigie et al. 2010; Scholte et al. 2025).

The Niokolo‐Koba National Park (NKNP), located in southeastern Senegal, is one of the largest remaining protected areas in West Africa (UNESCO World Heritage Centre 2025). It has been listed as a UNESCO World Heritage Site since 1981 due to its ecological importance (UNESCO World Heritage Committee 1981). The park contains a mosaic of savannahs, gallery forests and wetlands, and historically supports a wide range of mammal species, including the giant eland ( *Taurotragus derbianus* ), chimpanzees ( *Pan troglodytes verus* ), lions ( *Panthera leo leo* ), leopards ( *Panthera pardus* ), and elephants (*Loxodonta* sp.), likely *L. cyclotis* although the taxonomy is uncertain due to possible hybridisation between *L. africanus* and *L. cyclotis* (Dupuy 1971; Kuhner et al. 2025). Over the past decades, NKNP has experienced a range of pressures, including poaching, livestock grazing, and the planned construction of a dam on the Gambia and Niokolo Rivers upstream of the park, which have severely threatened its water regime. In 2007, these challenges led to its inclusion on the UNESCO List of World Heritage in Danger (UNESCO World Heritage Committee 2007). Following increased conservation and monitoring efforts, the park was removed from the list in 2024 (UNESCO World Heritage Committee 2024; Houéhounha et al. 2025). Nonetheless, a range of threats to the park's biodiversity persist, including the progressive drying of previously perennial water bodies due to invasive species such as *Mimosa pigra* , bush encroachment, illegal logging and grazing, mining, and associated water pollution (UNESCO World Heritage Committee 2024). These changes may have serious implications for wildlife, particularly for the park's water regime during the dry season, when access to water becomes a limiting factor (Houéhounha et al. 2025; UNESCO World Heritage Committee 2025).

In this study, we analysed camera‐trap data collected to monitor predator presence near Simenti, a ranger post in the northwestern sector of NKNP (Ohrndorf et al. 2025a, 2025b). We explored these data to assess species diversity and spatiotemporal activity patterns of large and medium‐sized mammals across habitat types and seasons. Specifically, we assessed which species were present and how frequently they were captured by the cameras year‐round. We determined diversity indices and local species detection rates to examine whether and how species detections varied across locations. Further, we investigated potential seasonal variation in habitat use across species. We then estimated temporal activity patterns of frequently detected species across the entire study site and across habitat types to gain insights into the spatiotemporal structure of habitat use of the local mammal community. We aim to provide high‐resolution, up‐to‐date information on mammal diversity and activity patterns in this section of NKNP, supporting ongoing conservation efforts and contributing to a broader understanding of mammal ecology in the local habitat mosaic.

## 2 Material and Methods

### 2.1 Study Site

**FIGURE 1:**
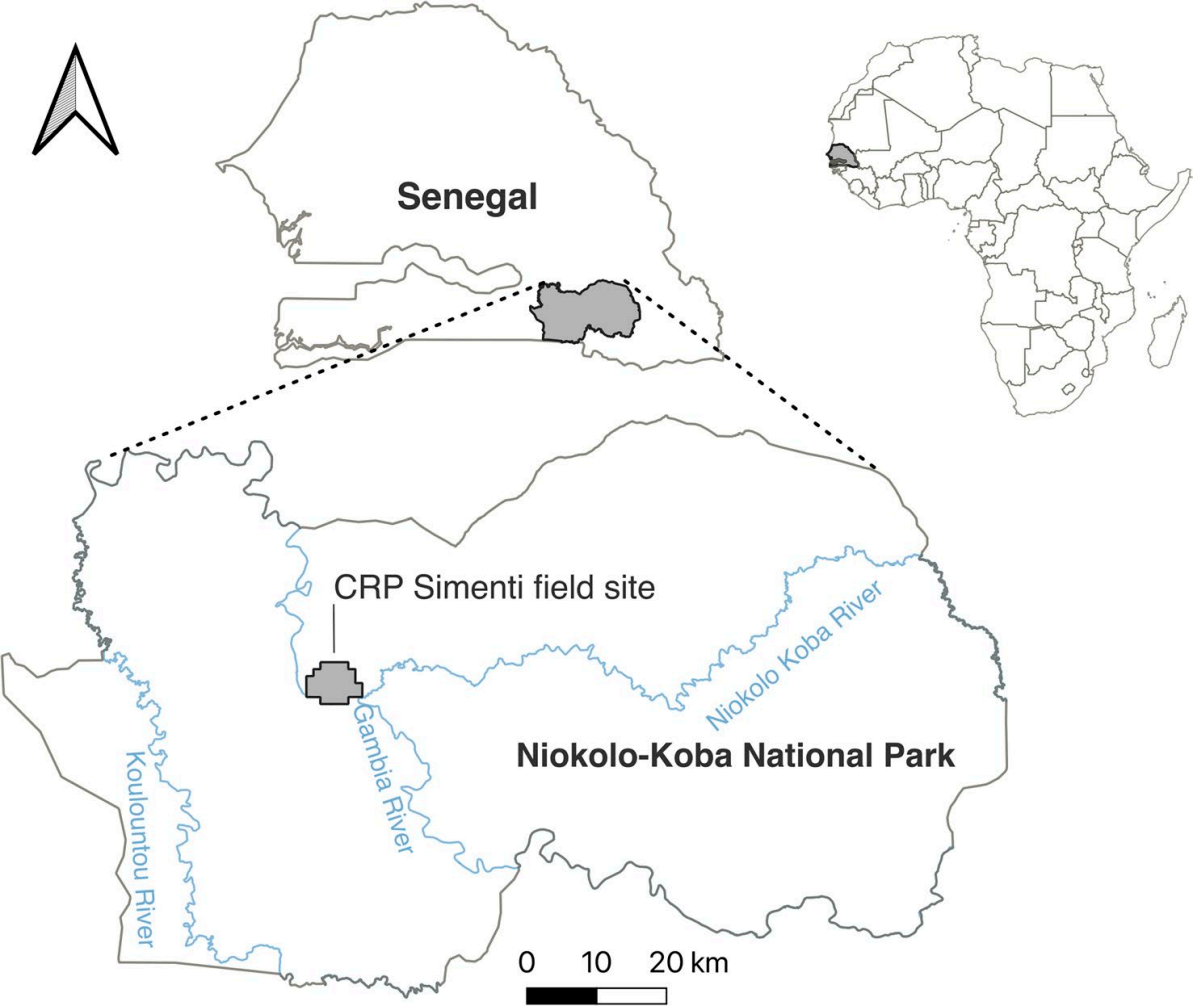
Location of the study site within the Niokolo‐Koba National Park in southeast Senegal.

Data collection for this study took place at the Centre de Recherche de Primatologie (CRP) long‐term field site in Simenti (Fischer et al. 2017). The field site is located in southeast Senegal within the NKNP next to the Gambia River (Figure 1). The site lies within the Sudanian and Sahelo‐Sudanian climatic zones and is characterised by pronounced seasonality and considerable seasonal variability of rainfall (Arbonnier 2002). Average annual precipitation in Simenti is approximately 950 mm, with the wet season typically lasting from June to October. May and mid‐October are transitional periods with little rainfall (Arbonnier 2002). In 2022, temperatures at the study site ranged from 11.6°C to 43.2°C during the dry season and from 21.5°C to 38.4°C during the wet season, as measured using a digital thermometer (Eurochrom ETH 5500 Thermometer/Hygrometer) positioned outdoors at the field site but protected from direct sunlight and precipitation. The vegetation is classified as a mosaic of grasslands, wooded savannahs, and gallery forests along streams and other perennial water bodies (Arbonnier 2002; Burgess et al. 2004).

### 2.2 Camera‐Trap Setup

The data analysed in this study were collected using unbaited, motion‐triggered camera‐traps deployed from February 2022 to March 2023. To ensure systematic sampling across a wide area, the cameras were positioned in a grid with approximately one camera per km^2^, resulting in 37 sampling points. In total, 45 camera‐traps were available (33 SECACAM Pro Plus, 1 Braun Scouting Cam Black 1300, 3 TEC.BEAN SG‐009, 3 Coolife Trail Camera, and 5 DÖRR Snap Shot Limited 5.0S). Of these, 37 were deployed simultaneously across sampling points, while the remaining eight served as backup in case of malfunction. All cameras, except one, were mounted on trees at approximately 0.5–0.6 m above ground level, facing visible animal trails. One camera, located on an open laterite airfield, could not be placed near an evident animal trail. The true camera‐trap locations deviated on average by 16.9 m from the planned grid points, with only two exceeding a deviation of 50 m (for the approximate camera positions within the grid see Figure 3). To ensure continuous data collection, we replaced the batteries and memory cards of all cameras monthly. During the wet season, we cleared a five‐metre radius around each camera of grass and weeds to maintain unobstructed visibility and prevent fire damage. All cameras were programmed to take three consecutive pictures per trigger event, with a 1‐min interval between triggers to avoid immediate retriggering.

### 2.3 Habitat Types

Habitat types in the study area were determined using supervised habitat classification based on multispectral Landsat 5 TM imagery from 28 November 2010 (Klapproth 2010). The habitat classification distinguished six physiognomic habitat types (gallery forest/forest, savannah woodland, tree/shrub savannah, grass savannah, temporary wetlands, and wetlands) and focused on 158 km^2^ in the Simenti region. We aggregated these six habitat types into three overall classes (gallery forest/forest = *forest*; savannah woodland, tree/shrub savannah, grass savannah = *savannah*; temporary wetlands, wetlands = *wetland*). Of our 37 camera‐trap sampling points, 10 were in forest habitats, 25 in savannah habitats, and 2 in wetlands.

### 2.4 Data Analysis

#### 2.4.1 Global Diversity

We uploaded all pictures to the platform ‘Agouti’ for a first, AI‐assisted, species identification (Casaer et al. 2019). We then reviewed all observations annotated by the AI to confirm or modify its annotations. We sampled 13,080 camera‐trap days between February 2022 and March 2023 (per sampling point: 225–390 days; mean = 353, SD = 38.8). We recorded a total of 52,585 events, containing 25,990 animal observations, 24,365 ‘blank’ observations (i.e., moving grass, bushfires), 732 vehicles (i.e., cars, planes, helicopters), 477 humans, and 1021 unidentified observations (unidentifiable animals, or shapes that were not clearly animals or blank). The platform ‘Agouti’ automatically groups images taken less than 120 s apart into sequences, which we considered detection events. We treated sequences involving different species or separated by more than 120 s apart as separate events. As all cameras except one were positioned along visible animal trails, we assume that detections separated by more than 120 s were unlikely to represent the same individual remaining within the camera's field of view. In cases where a camera was clearly retriggered repeatedly by the same individual or group, we adjusted events manually.

#### 2.4.2 Species Accumulation Curves

To assess whether our sampling effort adequately captured the species present at the study site, we calculated species accumulation curves for both time and space using the *vegan* package version 2.7‐2 (Oksanen et al. 2025) in R version 4.4.2 (R Core Team 2024). We used the function *specaccum* with 1000 random permutations (without replacement) of the observed data to estimate the mean accumulation curves and their associated variation. We further calculated temporal and spatial sampling coverage Q (Fagen and Goldman 1977) as follows:

$$Q_{\text{Temporal}}=1-\frac{N1_{\text{Temporal}}}{I_{\text{Temporal}}}$$

$$Q_{\text{Spatial}}=1-\frac{N1_{\text{Spatial}}}{I_{\text{Spatial}}}$$

Here, *N*1_Temporal_ is the number of species recorded only once across all camera events, and *I*_Temporal_ is the total number of camera events. *N*1_Spatial_ is the number of species that only occurred at one sampling point of the grid, and *I*_Spatial_ is the sum of the number of sites each species was detected. Values of Q close to 1 indicate high sampling completeness and a low probability of detecting additional species with further temporal (*Q*_Temporal_) or spatial (*Q*_Spatial_) coverage.

#### 2.4.3 Local Diversity

For each species, we calculated the relative abundance index (RAI), defined as the number of detections per 100 camera trap days. For each sampling point, we determined the local species richness (S) and the total number of animal sighting events per 100 days. We then calculated the evenness (J) for each sampling point as $J=\frac{H}{\log\left(S\right)}$, where *H* is the Shannon diversity index, and *S* is the species richness detected at a given site. We used evenness as the primary measure to compare diversity across sites, as it standardises the distribution of relative abundances among species and allows comparison among sampling points with differing species richness. To further characterise the spatial variation in species occurrence, we calculated the naïve occupancy, that is, the proportion of sampling sites at which a species was detected, for each species as an indicator of its spatial distribution across the study area. We then quantified the detection rates for each species across the three habitat types within the camera‐trapping grid to assess patterns of habitat use. To examine potential seasonal differences in species diversity and detection rates, we classified all sightings from 1 June to 31 October as ‘wet season’ and from 1 November to 31 May as ‘dry season’. We then calculated local species richness, number of animal sighting events per 100 days and evenness per season and sampling site.

To assess differences in animal sighting rates and diversity measures between the three habitat types and seasons, we fitted separate models for each response variable (species richness, animal sighting rate, evenness) with habitat type, season, and their interaction as the predictors using R. For species richness and animal sighting rates, we fitted two generalized linear mixed models with a Poisson error structure and log link function (McCullagh and Nelder 1989) using the function *glmmTMB* of the *glmmTMB* package version 1.1.14 (Brooks et al. 2017). For evenness, we fitted a generalized linear mixed model with a beta error structure and logit link function (McCullagh and Nelder 1989) using the *glmmTMB* package version 1.1.14 (Brooks et al. 2017). We included the log of the sampling effort per site and season as an offset term and the sampling site as a random effect in all models. For all models, we checked whether the assumption of no overdispersion was met. With dispersion parameters of 0.44, 0.57, and 0.87, respectively, the responses exhibited mild to moderate underdispersion given the models. Underdispersion indicates less variability than expected under the assumed error distribution and is generally considered less problematic than overdispersion, as it tends to result in conservative rather than anti‐conservative inference (Bolker et al. 2025). To determine the significance of the predictors, we used likelihood ratio tests (LRT) comparing the full model to reduced models in which each fixed effect was sequentially removed (full–reduced model comparisons; Dobson 2002). As the interaction terms were not significant in either model (Table S1), we excluded them and refitted the final models, including only the main effects of habitat and season.

For all models, we assessed stability by dropping each sampling point from the data one at a time and comparing the estimates from models fitted to these subsets to those from the full dataset. All models were of good stability (see results). We obtained confidence intervals of model estimates and fitted values using parametric bootstrapping (*N* = 1000; function *simulate* of the package *glmmTMB*). To assess potential spatial autocorrelation in our data, we plotted the difference in residuals against the spatial distance between two sampling sites. This allowed us to visually evaluate whether residuals at spatially closer sites were related, indicating spatial autocorrelation. We further assessed Pearson correlation coefficients between residual difference and spatial distance. Neither the visual inspection nor the Pearson correlation coefficients indicated strong spatial autocorrelation in the data (Figure S1).

To further assess species‐specific differences in detection rates across habitats and seasons, we fitted separate generalized linear mixed models following the same approach as for the models of species richness and animal sighting rates. A detailed description of the modelling approach, model structure and results can be found in Tables S3 and S4. Because most species had relatively low detection rates across habitat: season combinations, model estimates were unstable, particularly for wetland: season combinations. These results should therefore be interpreted with caution and considered as descriptive patterns rather than robust statistical inference.

#### 2.4.4 Activity Patterns

For species with 25 or more records across the camera‐trapping grid, we quantified diel activity patterns from camera‐trap detections using the *overlap* package version 0.3.9 (Meredith et al. 2024) in R version 4.4.2 (R Core Team 2024). For each species, we extracted the timestamps of independent detections, converted them to radians, and fitted nonparametric circular kernel density functions to estimate activity distributions. We derived average sunrise and sunset times at the study site using the *suncalc package* (Thieurmel et al. 2019). We transformed clock times to solar times to account for seasonal shifts in day length and uneven sampling across dates, using the average‐anchoring approach of Vazquez et al. (2019). Thus, activity is expressed relative to average sunrise and sunset times across the study period, weighted by the number of records per day. We then used the function *densityPlot* to visualise diel activity patterns for each species. To further investigate whether species use the three habitat types differently throughout the day, we estimated each species' activity distribution for each habitat type separately. We present these patterns descriptively, as formal species‐specific comparisons were not feasible for many habitat: season combinations due to limited sample sizes.

## 3 Results

### 3.1 Global Diversity

In total, we recorded 37 medium to large mammal species in the study area, including several of conservation concern according to the IUCN Red List (2026) (Table 1).

**TABLE 1:** Overview of species identified from February 2022 to March 2023 in Simenti, Senegal, across 37 sampling points and 13,080 camera‐trap days. Indicated is the total number of records, the number of records per habitat type, the relative abundance index (RAI) per 100 camera‐trap days across the entire grid, the range of the number of individuals detected per animal sighting event, and the proportion of locations at which a species was recorded (naïve occupancy) across the camera‐trapping grid. The IUCN Conservation status (IUCN 2026) for each species is included as a superscript.

Species	English name	Records				RAI	Individuals per event	Naïve occupancy
		Total	Forest	Savannah	Wetland			
**Tubulidentata**								
*Orycteropus afer* ^LC^	Aardvark	73	15	58		0.56	1–2	0.43
**Primates**								
*Papio papio* ^NT^	Guinea baboon	3302	1972	1225	105	25.24	1–54	1.00
*Erythrocebus patas* ^NT^	Patas monkey	229	31	195	3	1.75	1–8	0.68
*Chlorocebus sabaeus* ^LC^	Green monkey	543	396	140	7	4.15	1–11	0.59
*Galago senegalensis* ^LC^	Senegal galago	59	4	55		0.45	1–2	0.24
**Rodentia**								
*Hystrix cristata* ^LC^	Crested porcupine	272	180	91	1	2.08	1–3	0.51
**Lagomorpha**								
*Lepus* sp.^LC^	African savannah hare	12	1	7	4	0.09	1–2	0.19
**Carnivora**								
*Lupulella adusta* ^LC^	Side‐striped jackal	101		100	1	0.77	1–2	0.43
*Lycaon pictus* ^CR^	African wild dog	4		4		0.03	1	0.05
*Aonyx capensis* ^NT^	African clawless otter	3	3			0.02	1	0.05
*Mellivora capensis* ^LC^	Honey badger	12	9	3		0.09	1–2	0.22
*Panthera l. leo* ^CR LD^	African lion	25	6	18	1	0.19	1–4	0.38
*Panthera pardus* ^EN LD^	Leopard	82	67	10	5	0.63	1	0.38
*Leptailurus serval* ^LC^	Serval	22		21	1	0.17	1–2	0.24
*Felis lybica* ^LC^	African wildcat	2	1	1		0.02	1	0.05
*Crocuta crocuta* ^LC^	Spotted hyaena	133	38	94	1	1.02	1–3	0.76
*Civettictis civetta* ^LC^	African civet	73	39	33	1	0.56	1–2	0.41
*Genetta genetta* ^LC^	Common genet	89		87	2	0.68	1	0.30
*Genetta pardina* ^LC^	Pardine genet	359	303	20	36	2.74	1–2	0.32
*Atilax paludinosus* ^LC^	Marsh mongoose	2	2			0.02	1	0.03
*Herpestes ichneumon* ^LC^	Egyptian mongoose	21	9	12		0.16	1–2	0.24
*Herpestes sanguineus* ^LC^	Common slender mongoose	1	1			0.01	1	0.03
*Ichneumia albicauda* ^LC^	White‐tailed mongoose	381	145	229	7	2.91	1–2	0.54
*Mungos mungo* ^LC^	Banded mongoose	143	79	64		1.09	1–14	0.46
*Mungos gambianus* ^LC^	Gambian mongoose	35	1	34		0.27	1–8	0.30
**Certartiodactyla**								
*Phacochoerus africanus* ^LC^	Common warthog	1589	330	903	356	12.15	1–9	0.97
*Potamochoerus porcus* ^LC^	Red river hog	32	6	26		0.24	1–7	0.38
*Hippopotamus amphibius* ^VU^	Hippopotamus	36	28	4	4	0.28	1–2	0.30
*Syncerus caffer* ^NT^	African buffalo	20	5	15		0.15	1–4	0.30
*Tragelaphus scriptus* ^LC^	Bushbuck	11,056	4867	5614	575	84.53	1–9	1.00
*Sylvicapra grimmia* ^LC^	Common duiker	290		290		2.22	4	0.51
*Cephalophorus rufilatus* ^LC^	Red‐flanked duiker	396	240	124	32	3.03	1–3	0.76
*Ourebia ourebi* ^LC^	Oribi	513	3	508	2	3.92	1–4	0.49
*Kobus kob kob* ^VU^	Kob	1607	39	845	723	12.29	1–15	0.76
*Kobus ellipsiprymnus defassa* ^NT^	Waterbuck	1941	480	1017	444	14.84	1–10	0.97
*Alcelaphus buselaphus major* ^VU^	Western hartebeest	1		1		0.01	5	0.03
*Hippotragus equinus* (*koba*)^LC^	Roan antelope	1175	82	1069	24	8.98	1–11	0.89

Abbreviations: LC, least concern; NT, near threatened; VU, vulnerable; EN, endangered; CR, critically endangered (IUCN red list assessment); LD, largely depleted (IUCN green status assessment) (accessed November 2025).

### 3.2 Species Accumulation Curves

**FIGURE 2:**
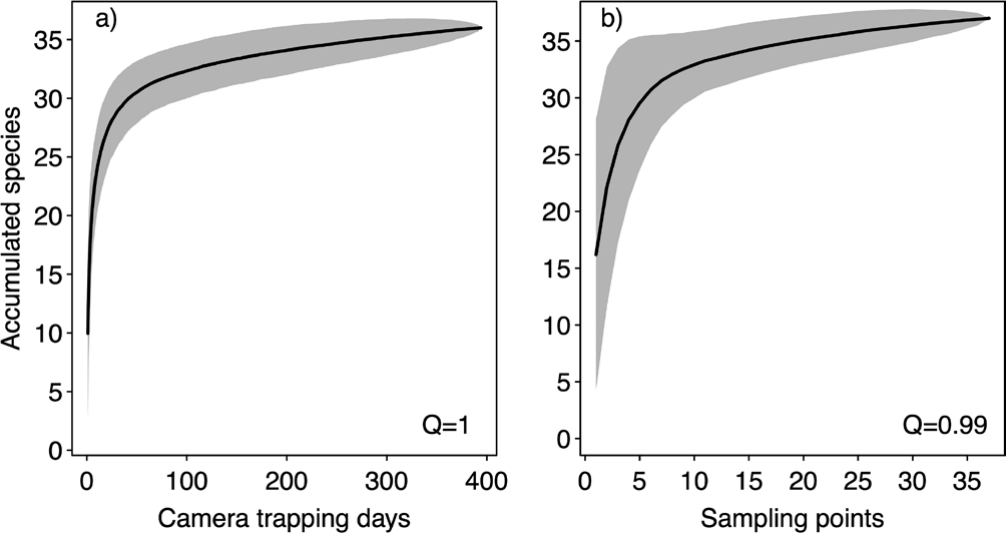
(a) Temporal and (b) spatial species accumulation curves. The black line depicts the estimated mean, and the grey shaded areas depict confidence intervals.

Species accumulation curves based on camera‐trap days and sampling points approached an asymptote (Figure 2). Values for Q were 1 and 0.99 for temporal and spatial sampling coverage, respectively, indicating that the sampling effort was sufficient to capture most medium‐ to large‐bodied species detectable by our setup.

### 3.3 Local Diversity

**FIGURE 3:**
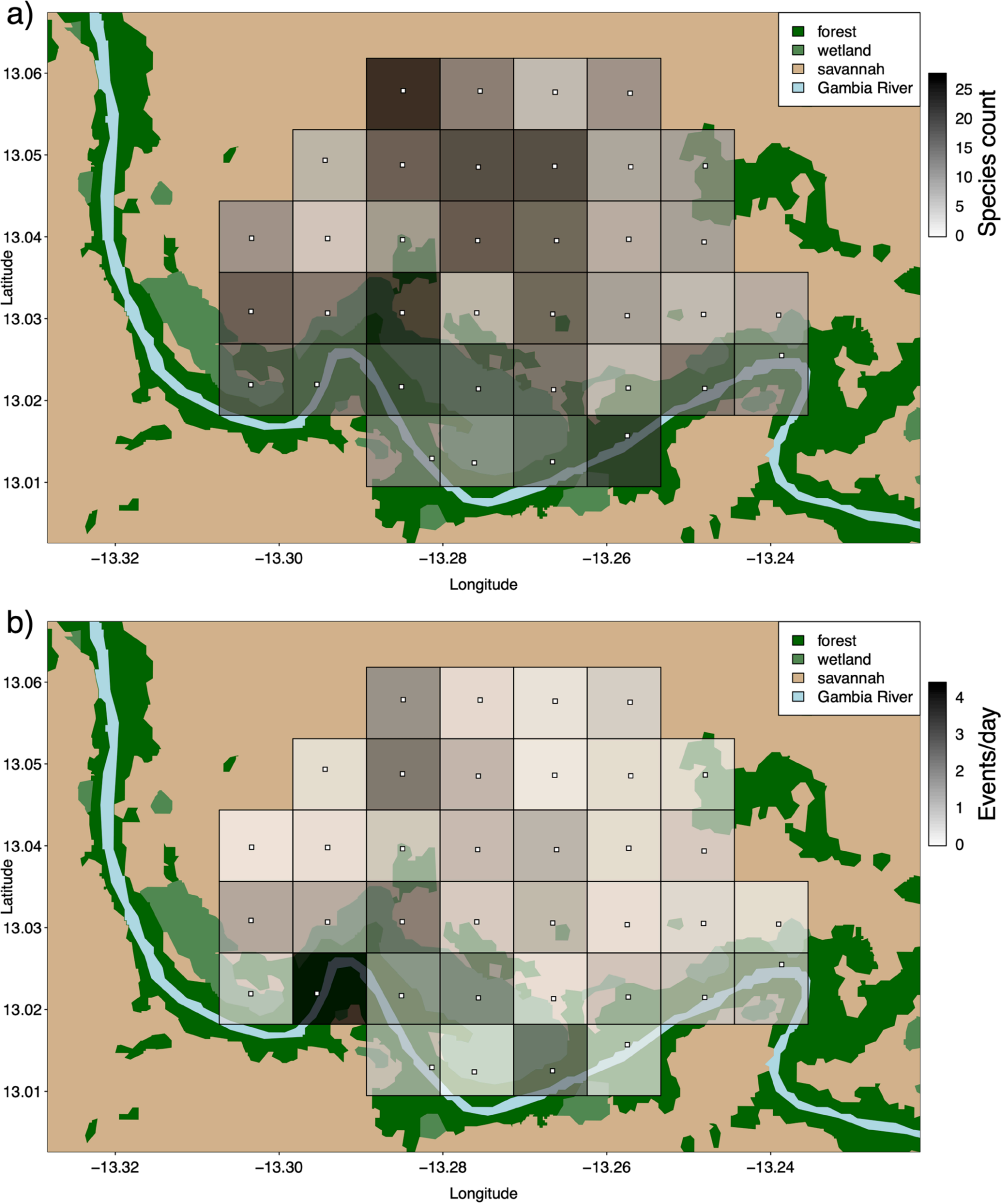
(a) Local species richness and (b) animal sighting events per day and sampling point (white squares, camera positions), both from February 2022 to March 2023 across the study site.

**FIGURE 4:**
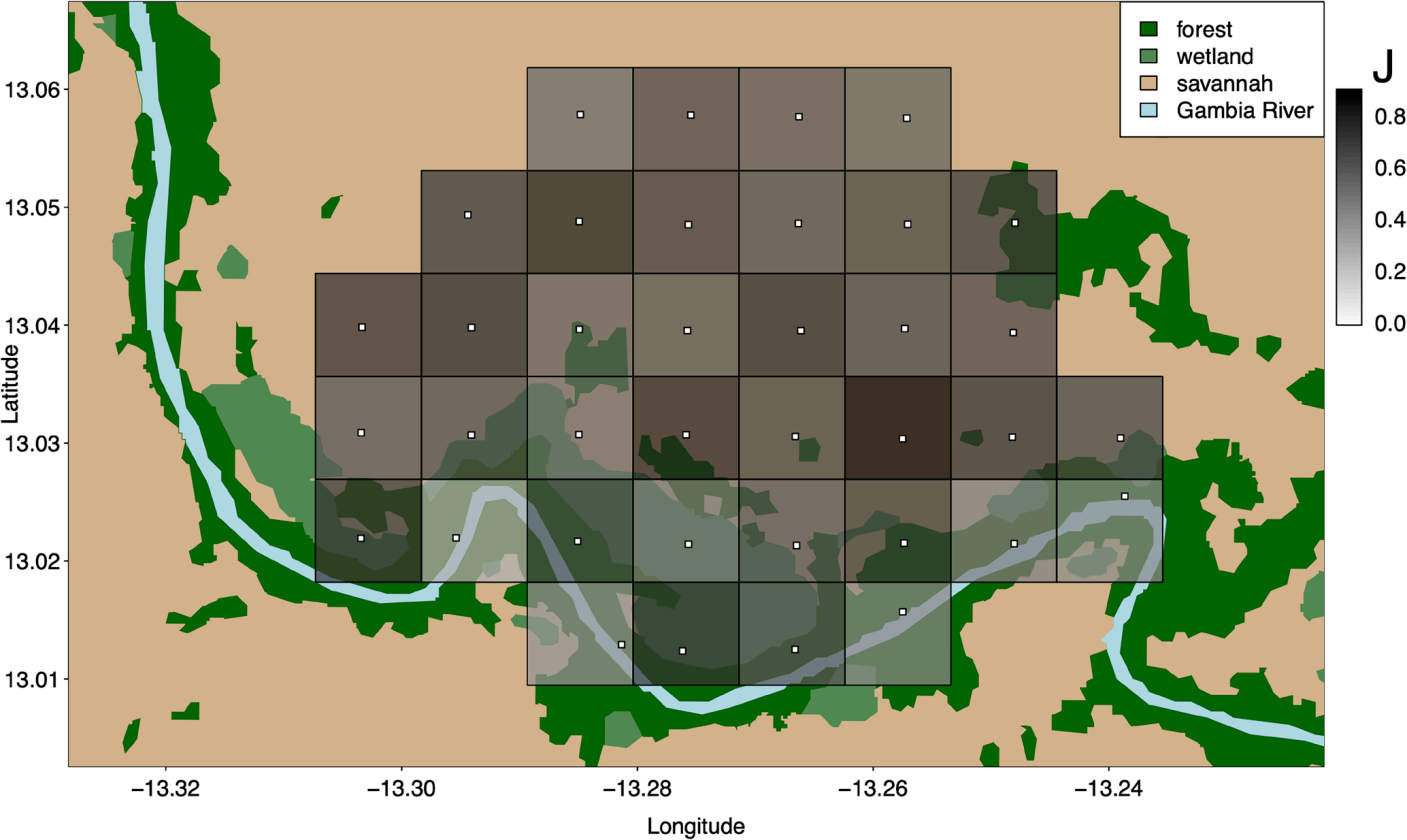
Local evenness values (J) calculated per sampling point (white squares, camera positions) from February 2022 to March 2023 across the study site.

The number of species recorded per sampling point ranged from 5 to 28, with a median of 16 (Figure 3a). The number of animal‐sighting events per day and per camera ranged from 0 to 6 (Figure 3b). Evenness values were moderate across the study site, ranging from 0.37 to 0.79 with a mean of 0.6, suggesting relatively uniform species distributions at most sites (Figure 4). As no model revealed significant habitat: season interactions (Table S1), we based further inference on the reduced models containing only the main effects of habitat and season. Species richness did not differ among habitat types (LRT: χ^2^ = 0.416, df = 2, *p* = 0.812) but was higher in the dry season than in the wet season (LRT: χ^2^ = 9.996, df = 1, *p* = 0.001) (Table 2). Habitat type (LRT: χ^2^ = 14.973, df = 2, *p* < 0.001) and season (LRT: χ^2^ = 26.245, df = 1, *p* < 0.001) were significant predictors of evenness across the study site. Evenness values were significantly higher at savannah sites than at forest sites, and higher during the wet season compared to the dry season (Table 2). Neither habitat type (LRT: χ^2^ = 0.579, df = 2, *p* = 0.749) nor season (LRT: χ^2^ = 1.468, df = 1, *p* = 0.226) predicted the number of animal sightings per day. Given the small number of wetland sampling sites (*n* = 2), we do not interpret differences between wetlands and other habitat types.

**TABLE 2:** Model results on species richness, animal sighting rates, and evenness; estimates, standard errors, confidence intervals, likelihood ratio tests, significance tests, and range of estimates derived from dropping each sampling site one at a time.

Model	Term	Estimate	SE	CI_Lower_	CI_Upper_	ꭓ^2^	df	*p*	Min	Max
Species richness	Intercept	−2.763	0.113	−2.996	−2.543				−2.802	−2.703
	habitat.savannah^a^	0.010	0.130	−0.220	0.273	0.416	2	0.812	−0.046	0.045
	habitat.wetland^a^	−0.158	0.273	−0.709	0.345				−0.475	0.110
	season.wet^b^	0.211	0.066	0.083	0.340	9.996	1	**0.001**	0.198	0.231
Sighting rate	Intercept	−0.121	0.363	−0.848	0.590				−0.318	0.224
	habitat.savannah^a^	−0.298	0.417	−1.082	0.537	0.579	2	0.749	−0.639	−0.090
	habitat.wetland^a^	0.008	0.857	−1.669	1.618				−1.191	1.111
	season.wet^b^	−0.218	0.178	−0.573	0.127	1.468	1	0.226	−0.270	−0.161
Evenness	Intercept	−5.307	0.122	−5.556	−5.085				−5.393	−5.247
	habitat.savannah^a^	0.582	0.136	0.335	0.841	14.973	2	**< 0.001**	0.514	0.672
	habitat.wetland^a^	0.401	0.282	−0.095	0.916				0.133	0.679
	season.wet^b^	0.544	0.090	0.355	0.715	26.245	1	**< 0.001**	0.516	0.577

^a^ Variable dummy coded with the reference level being forest. The indicated test refers to the overall effect of habitat derived from the full‐null model comparisons.

^b^ Variable dummy coded with the reference level being dry season. The indicated test refers to the overall effect of season derived from the full‐null model comparison.

Naïve occupancy of species ranged from 0.03 to 1, where 0.03 indicates occurrence at only one out of 37 sampling sites, and 1 indicates occurrence at all sites. Guinea baboons ( *Papio papio* ) and northern bushbucks ( *Tragelaphus scriptus* ) were most widely distributed (1), followed by waterbucks ( *Kobus ellipsiprymnus defassa* ), northern warthogs (*Phacochoerus a. africanus*; 0.97), and by roan antelopes ( *Hippotragus equinus* ; 0.89). The least widely distributed species (0.03) were marsh mongooses (*Atilax paludinosus)*, slender mongooses (*Herpestes sanguineus*), and western hartebeests ( *Alcelaphus buselaphus major* ). These species were also among the least frequently recorded (1–2 total records). Maps showing the number of individuals detected per species and sampling site within 100 days of camera‐trapping are provided in Figures S2–S38. Patterns of habitat use varied between species. Fifteen out of 37 species were mostly detected in savannah habitats (e.g., side‐striped jackal [*Lupulella adusta*], common genet [ *Genetta genetta* ], common duiker [ *Sylvicapra grimmia* ]), while seven species were mostly detected in forests (e.g., *Papio papio* , *Panthera pardus* , and the pardine genet [ *Genetta pardina* ]) (Table 1). The remaining species were either detected too infrequently or showed no clear pattern of habitat use.

We observed varying patterns of seasonal habitat use across species. We only report seasonal differences in habitat use for species with at least 25 total records. For some species (e.g., *Papio papio* , green monkey [ *Chlorocebus sabaeus* ], crested porcupine [ *Hystrix cristata* ], banded mongoose [ *Mungos mungo* ]), habitat use did not seem to differ between seasons. For several other species, records were proportionally more frequent in forests during the dry season and in savannahs during the wet season (e.g., red‐flanked duiker (*Cephalophorus rufilatus*), African civet ( *Civettictis civetta* ), patas monkey ( *Erythrocebus patas* ), *Panthera pardus* ) (Table S2). Species‐specific models revealed a significant interaction of habitat and season only for *C. rufilatus* (LRT: χ^2^ = 35.873, df = 2, *p* < 0.001), indicating a shift from forest to savannah habitats in the wet season compared to the dry season (Table S4).

### 3.4 Activity Patterns

**FIGURE 5:**
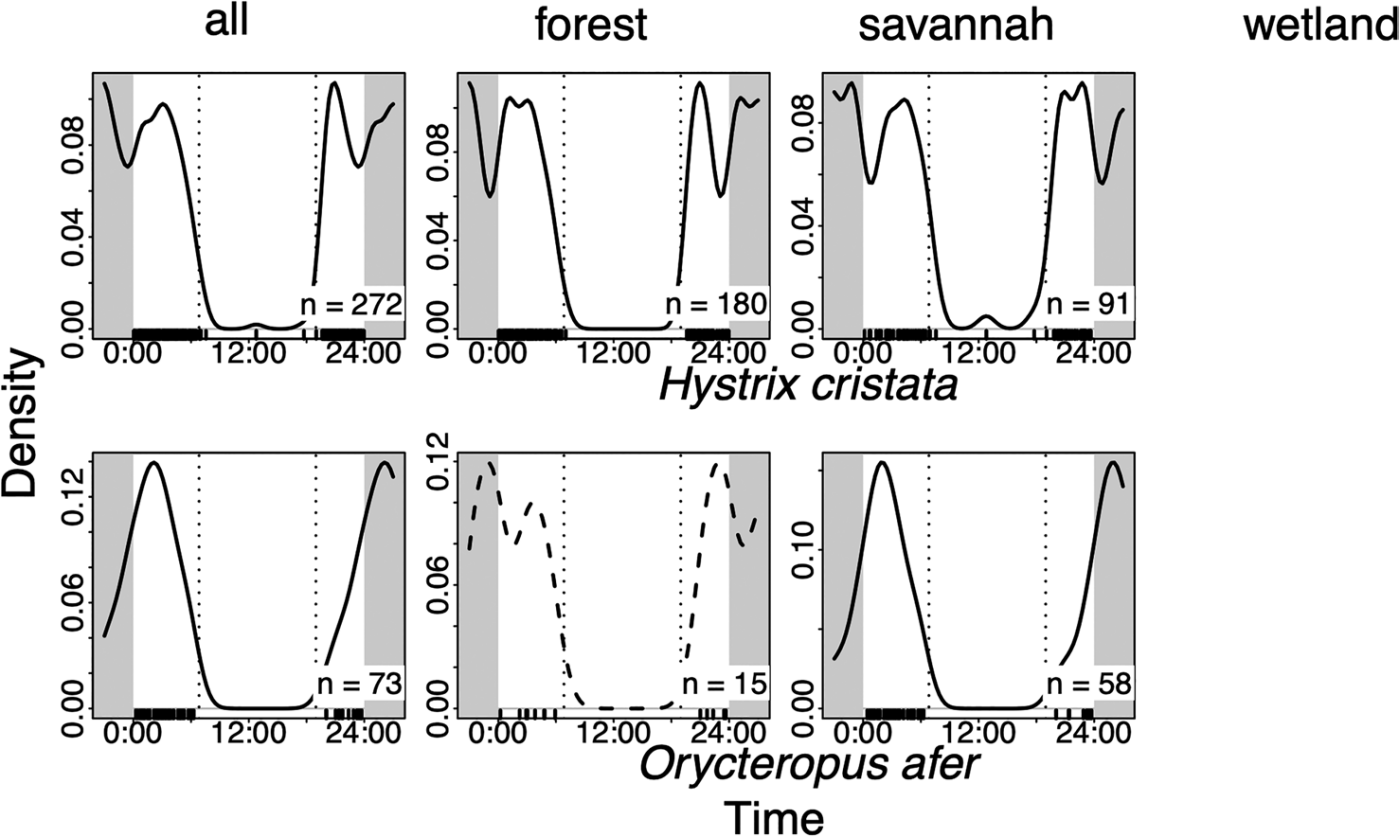
Diel activity patterns for *Hystrix cristata* and *Orycteropus afer* and respective sample sizes (*n*) across the study site (all) and by habitat type (forest, savannah, wetland). Dashed lines indicate low sample sizes (< 25 records). Blank panels are due to insufficient data coverage (only one or no sighting of a species in a given habitat type). Dotted lines indicate the average times of sunrise and sunset at the study site. The original observations are displayed as a rug along the timeline at the bottom of the plots.

**FIGURE 6:**
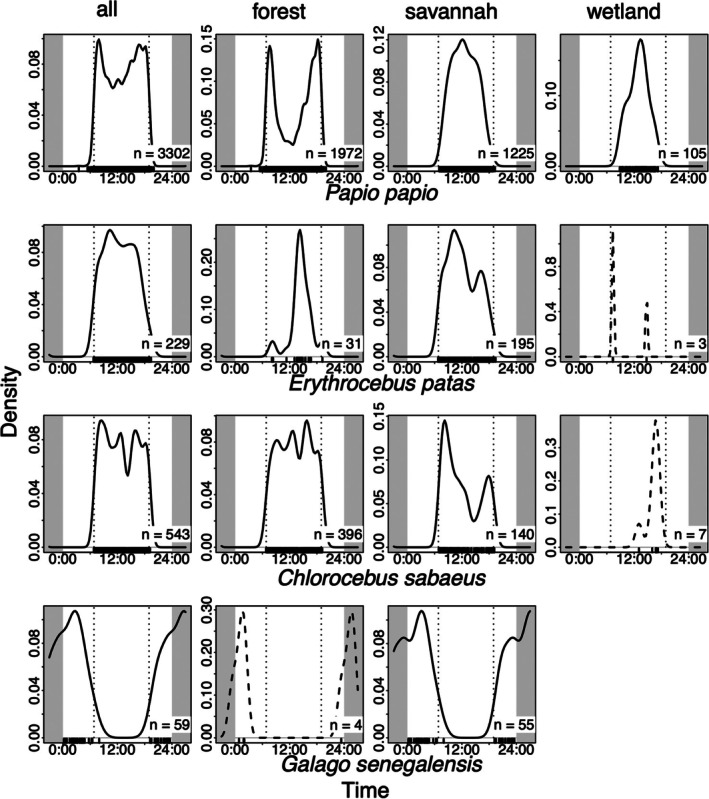
Diel activity patterns for the recorded primate species and respective sample sizes (*n*) across the study site (all) and by habitat type (forest, savannah, wetland). Dashed lines indicate low sample sizes (< 25 records). Blank panels are due to insufficient data coverage (only one or no sighting of a species in a given habitat type). Dotted lines indicate the average times of sunrise and sunset at the study site. The original observations are displayed as a rug along the timeline at the bottom of the plots.

**FIGURE 7:**
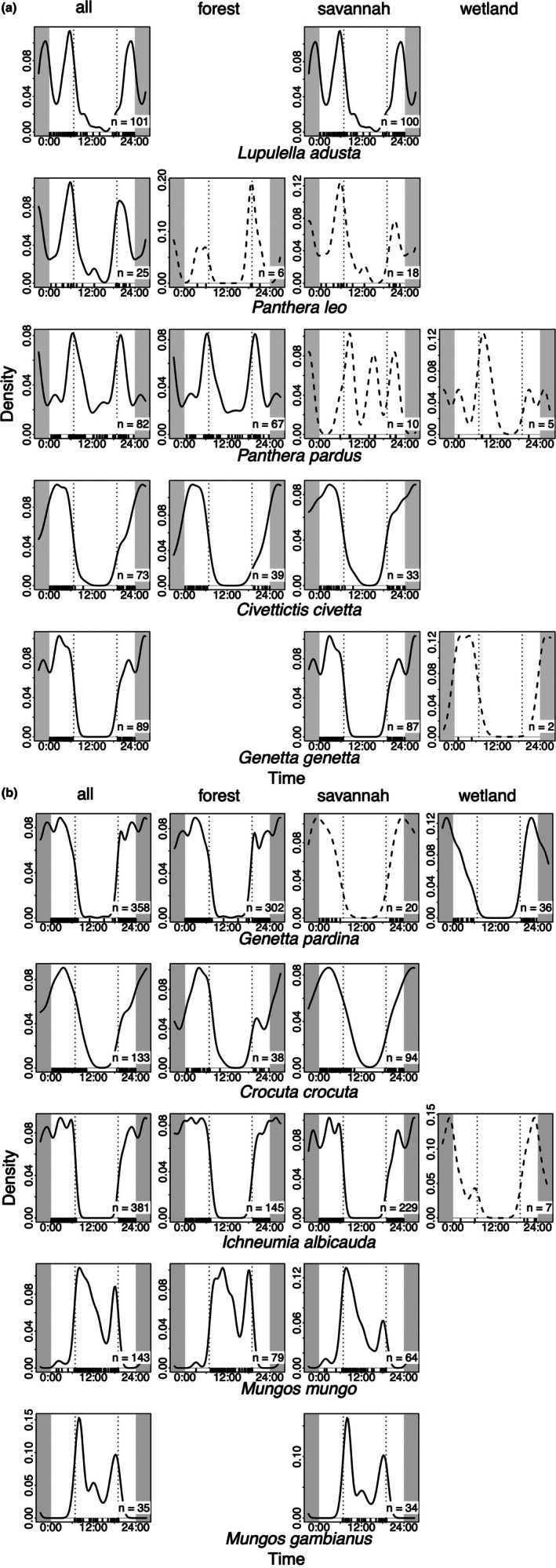
(a, b) Diel activity patterns of recorded carnivore species and respective sample sizes (*n*) across the study site (all) and by habitat type (forest, savannah, wetland). Dashed lines indicate low sample sizes (< 25 records). Blank panels are due to insufficient data coverage (only one or no sighting of a species in a given habitat type). Dotted lines indicate the average times of sunrise and sunset at the study site. The original observations are displayed as a rug along the timeline at the bottom of the plots.

**FIGURE 8:**
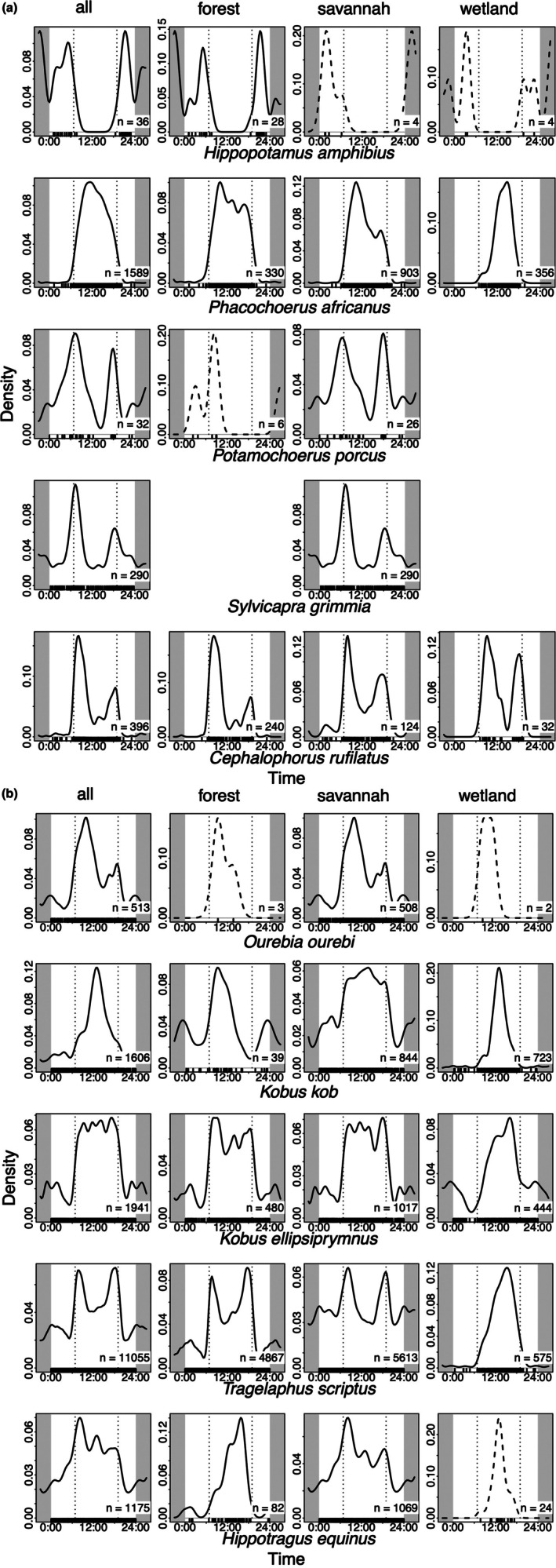
(a, b) Diel activity patterns of recorded certartiodactyl species and respective sample sizes (*n*) across the study site (all) and by habitat type (forest, savannah, wetland). Dashed lines indicate low sample sizes (< 25 records). Blank panels are due to insufficient data coverage (only one or no sighting of a species in a given habitat type). Dotted lines indicate the average times of sunrise and sunset at the study site. The original observations are displayed as a rug along the timeline at the bottom of the plots.

For 26 out of 37 detected mammal species, we were able to estimate diel activity patterns. Some species exhibited clearly nocturnal (e.g., *Hystrix cristata*, aardvark [ *Orycteropus afer* ] [Figure 5]), crepuscular (e.g., *Lupulella adusta, Panthera pardus* [Figure 7a,b]), or diurnal (e.g., *Papio papio, Phacochoerus africanus*; Figures 6 and 8a) activity patterns. For other species, particularly ruminants (e.g., *Tragelaphus scriptus* , *Hippotragus equinus* ; Figure 8b), diel activity patterns were more cathemeral, showing multiple peaks across day and night and a relatively high baseline of nocturnal activity.

Most species' activity distribution did not seem to differ between habitat types (Figures 5, 6, 7, 8). *Papio papio* exhibited higher levels of activity in forest habitats in the mornings and evenings, and in savannahs and wetlands during the day. Several large herbivores (kob [ *Kobus kob* ], *Tragelaphus scriptus* , *Hippotragus equinus* ) seemed to shift their activity from wetlands during the day to forests and savannahs during the night. Although very low sample sizes did not permit a reliable estimation of activity distribution, we included these panels (with dashed lines) to visualize data availability and distribution, but refrained from interpreting any patterns.

## 4 Discussion

### 4.1 Global Diversity

Our camera‐trap survey provides a comprehensive inventory of medium‐ to large‐sized terrestrial mammals in Niokolo‐Koba National Park. We documented 37 species, representing the majority of medium‐sized to large mammal species expected to occur in the Simenti area based on IUCN range maps (IUCN 2026). When considering only medium‐ to large‐sized mammals, our inventory largely aligns with both historical and more contemporary records for the region (Dupuy 1971; Kane 2014; Rabeil et al. 2018; Horion et al. 2024; Mirghani et al. 2025). Although small, arboreal, or aerial species fall outside the detection range of our setup, our study effectively captured the local terrestrial mammal assemblage, including several species of conservation concern.

Several of the park's flagship species, including chimpanzees ( *Pan troglodytes verus* ) and giant eland ( *Taurotragus derbianus* ), were not recorded in our study area, although they occur in other sectors of the park (Kane 2014; Rabeil et al. 2018; Horion et al. 2024). Their absence may reflect a combination of factors, including limited suitable habitat, floral and structural diversity, and the presence of large predators at our study site (Lindshield et al. 2019; Wessling et al. 2020; Stehlíková 2023). Other taxa expected to occur at low densities throughout the park, including caracals ( *Caracal caracal* ), Bohor reedbuck ( *Redunca redunca* ), African palm civet ( *Nandinia binotata* ), and rock hyraxes (*Procavia ruficeps*), were also not recorded but have been reported to occur in the southeastern sections of the park and surrounding areas (Kane 2014; Horion et al. 2024; Mirghani et al. 2025). Their absence may likewise reflect local ecological conditions, such as the absence of rocky cliffs or other habitat features important for these species.

Extremely rare species, such as elephants (*Loxodonta* sp.) and giant pangolin (*Smutsia gigantea*), were absent from our dataset. Recent records suggest that *Loxodonta* sp. may be limited to a single individual in the southern sector of the park (Panthera 2025), while the detection of *S. gigantea* in 2023 represents the first confirmed observation in 24 years (Ndiaye et al. 2024). These two species do not seem to occur in the Simenti area.

The Relative Abundance Index (RAI) values for most species at our study site are broadly comparable to those reported by Kane (2014), though with some notable differences. Carnivores (e.g., *Panthera leo, P* *. pardus* ) were detected up to 10 times more frequently in Kane's study. This is likely due to differences in sampling design. While Kane (2014) deliberately selected an area of particularly high large‐carnivore abundance identified in a pilot study, our camera‐trapping grid was designed to systematically cover the entire study area for the duration of a year.

### 4.2 Local Diversity

Our analyses revealed no significant difference in species richness between habitat types. The higher species richness recorded during the dry season compared to the wet season may partly reflect seasonal differences in detectability rather than true changes in community composition. Although a 5‐metre radius around each camera was cleared of grass and weeds during the wet season, denser vegetation likely reduced detection distances and obscured animals approaching from outside the cleared area. In addition, the broader availability of food and water during the wet season may allow animals to disperse more widely across the landscape, reducing the likelihood of detecting less common species. Evenness was higher in savannah than in forest habitats and higher in the wet season than in the dry season. The higher evenness observed during the wet season suggests a more balanced distribution of detections among species. This pattern may reflect the increased availability of water, palatable herbaceous vegetation, fruits, and invertebrates following the onset of rains, which may lead to a more even distribution of detections across species.

Animal sighting rates did not differ significantly between habitat types or seasons. While sighting rates at wetland sites were relatively high, direct comparisons with other habitats would be misleading, as wetlands were underrepresented in our setup and strongly affected by seasonal flooding and greater visibility. Nevertheless, wetlands and the Gambia River are the only perennial water sources in the area and are likely important landscape features for many species, particularly during the dry season (UNESCO World Heritage Centre 2025). Elevated animal sighting rates at some forest sites near the Gambia River, in contrast, appear to be driven by a subset of species disproportionately using these sites. In particular, certain parts of the gallery forest along the Gambia River exhibited this pattern, likely because Guinea baboons used tall trees as sleeping sites (Zinner et al. 2021; Ohrndorf et al. 2025b). Taken together, these patterns suggest that the landscape as a whole supports consistently high local diversity, but not all species use it homogeneously.

Several of the most frequently detected species (e.g., *Tragelaphus scriptus, Papio papio, Phacochoerus africanus, Kobus ellipsiprymnus, Hippotragus equinus*) were recorded across the entire study site, suggesting extensive use of the available habitat, whether due to high abundance, wide spatial movements, large home ranges, or a combination thereof. Other species showed more pronounced spatial clustering, likely reflecting habitat preferences or localised activity centres, potentially constrained by smaller home ranges or territories (e.g., Senegal galago [ *Galago senegalensis* ], *Genetta pardina, oribi* [ *Ourebia ourebi* ]).

Our assessment of habitat‐specific sighting frequencies suggested that several observed species shifted from forests in the dry season to savannahs in the wet season. This pattern may reflect a strategy to reduce exposure to sun and heat during the hottest months of the year and to remain close to surface water for drinking (Brain and Mitchell 1999; Terrien et al. 2011; Boyers et al. 2019). Gallery forests along the Gambia River and the Mare de Simenti provide access to the only perennial water sources. For grazers and folivores, and potentially for frugivores, omnivores, and insectivores, a shift toward savannah habitats in the wet season may also be driven by food availability. At the study site, leaves and fruits of many trees, as well as invertebrates and much of the palatable herbaceous vegetation in savannah habitats, typically only become available after the first rains of the wet season (Ohrndorf et al. 2025a). Note, however, that species‐specific analyses provided limited evidence for consistent habitat: season interactions. As such, the observed patterns should be interpreted as descriptive trends in habitat use that warrant further investigation rather than as evidence of consistent seasonal habitat shifts.

### 4.3 Activity Patterns

The temporal activity patterns estimated from our dataset largely reflect the expected diel behavioural patterns of the recorded species. Many species were clearly assignable to predominantly diurnal, nocturnal, or crepuscular activity patterns, indicating well‐defined temporal niches. Some diurnal species (e.g., *Mungos mungo* , Gambian mongoose (*M. gambianus)*, *Papio papio* ) showed a noticeable decline in detections during midday and early afternoon hours, potentially reflecting behavioural thermoregulation in response to high temperatures, particularly in open habitats (Dzingwena et al. 2025).

Other species showed more cathemeral activity patterns. Several large herbivores (e.g., *Tragelaphus scriptus* , *Hippotragus equinus* , *Kobus ellipsiprymnus* ) exhibited moderate to high activity levels throughout the 24‐h cycle, consistent with broad species‐level descriptions that report flexible activity patterns across the day (Kingdon et al. 2013). Some species deviated slightly from expectations. *Panthera leo* exhibited a more crepuscular activity pattern than reported for the region (Gueye et al. 2024; Horion et al. 2024), though this might be influenced by the small number of lion detections in our dataset (*n* = 25). *Panthera pardus* and *Lupulella adusta* showed more daytime activity than is commonly reported, a pattern often associated with low levels of anthropogenic disturbance (Kingdon et al. 2013; Gaynor et al. 2018). Increased levels of diurnal activity have also been reported for leopards at several densely forested study sites (Havmøller et al. 2020; van der Hoek et al. 2023; Versavaud et al. 2026), where they have been attributed to reduced interspecific competition and a lower risk of kleptoparasitism (Hayward and Slotow 2009; Havmøller et al. 2020). However, these mechanisms are unlikely to explain the patterns observed at our study site, where lions and hyenas are relatively common (Horion et al. 2024).

When examining species' activity distributions across the three habitat types separately, we found that some species appeared to shift their habitat use throughout the day. *Papio papio* , in particular, showed a pronounced bimodal pattern of forest use in the early mornings and late afternoons to evenings, with increased use of savannah and wetland habitats during the day. This pattern likely reflects the availability of suitable sleeping trees in gallery forests, which are heavily used by baboons, as well as the spatial segregation of sleeping sites and most daytime foraging areas (Zinner et al. 2021; Ohrndorf et al. 2025a, 2025b). *Kobus kob* , *Tragelaphus scriptus* , and *Hippotragus equinus* used wetland habitats mostly during the day, shifting to savannah and forest habitats at night. This shift may be driven by a preference for habitats offering greater cover during periods of increased vulnerability to predation (Bonnot et al. 2013; Burkepile et al. 2013; Anderson et al. 2016). Most recorded species, however, did not show a clear pattern of habitat use across the day, or lacked sufficient data to reliably assess habitat‐specific activity distributions.

While habitat characteristics and seasonal variation in resource availability likely contribute to many of the spatiotemporal patterns observed in this study, intra‐ and interspecific interactions may also play an important role. Reproductive opportunities, competition, kleptoparasitism, or predation risk may influence when and where animals are active, and whether they avoid or seek out certain areas in the landscape (Cusack et al. 2017; Frey et al. 2017; Nakazawa 2023; Grabowski et al. 2024). Future investigations of temporal and spatial overlap among sympatric species could provide additional insight into the mechanisms that shape the spatiotemporal dynamics of the local species assemblage.

## 5 Conclusion

Our camera‐trap survey provides high‐resolution, up‐to‐date information on the community of medium‐ to large‐sized mammals in the Simenti region of NKNP, documenting a species assemblage broadly consistent with historical and contemporary records. Evenness values were moderate to high across the landscape, and even areas with lower diversity appeared to be heavily used by certain species, as indicated by elevated animal sighting rates. The recorded species appeared to use the local habitat mosaic relatively uniformly, highlighting the ecological importance of all habitat types. A descriptive assessment of habitat‐specific sighting frequencies suggests potential seasonal shifts in habitat use for several species, likely indicating strategies for thermoregulation and/or varying levels of food availability across habitats and seasons. Diel activity patterns were consistent with expectations for the observed species. Some species exhibited differential use of the available habitats throughout the day. Altogether, our results provide a useful reference for future studies and monitoring efforts and help characterise the local species assemblage of the Simenti area within the larger NKNP ecosystem.

## Author Contributions

**Lisa Ohrndorf:** conceptualization (equal), data curation (lead), formal analysis (equal), investigation (lead), visualization (lead), writing – original draft (lead), writing – review and editing (equal). **Augustin Brouillet:** data curation (equal), formal analysis (equal), visualization (supporting), writing – original draft (supporting), writing – review and editing (equal). **Annika M. Zuleger:** formal analysis (equal), visualization (supporting), writing – original draft (supporting), writing – review and editing (equal). **Ndiouga Diakhaté:** investigation (supporting), writing – review and editing (equal). **Djibril Coly:** investigation (supporting), writing – review and editing (equal). **Chérif Younousse Kéba Camara:** investigation (supporting), writing – review and editing (equal). **Amadou Bamba Diedhiou:** investigation (supporting), writing – review and editing (equal). **Irene Gutiérrez Díez:** investigation (supporting), writing – review and editing (equal). **Julia Fischer:** conceptualization (equal), funding acquisition (equal), supervision (supporting), writing – review and editing (equal). **Dietmar Zinner:** conceptualization (equal), funding acquisition (equal), supervision (lead), writing – original draft (supporting), writing – review and editing (equal).

## Funding

This research was supported by the Deutsche Forschungsgemeinschaft (DFG, German Research Foundation), Grant/Award Number: 254142454/GRK 2070. This publication was supported by the Leibniz Association through funding for the Leibniz ScienceCampus Primate Cognition (W45/2019 Strategische Vernetzung).

## Ethics Statement

The data presented in this study were obtained exclusively through non‐invasive camera‐trapping.

## Conflicts of Interest

The authors declare no conflicts of interest.

## Supporting information


**Figure S1:** Relationship between pairwise spatial distance and pairwise residual difference for (a) species count, (b) number of sightings, and (c) evenness. Each point represents a unique pair of sampling sites (*n* = 666). Spatial autocorrelation would result in a clear clustering of residual differences with spatial distance. Pearson correlation coefficients were low (species count: *r* = 0.045; sightings: *r* = −0.010; evenness: *r* = 0.030), suggesting little to no spatial autocorrelation.
**Figure S2:** Number of individuals of *Orycteropus afer*.
**Figure S3:** Number of individuals of *Papio papio*.
**Figure S4:** Number of individuals of *Erythrocebus patas*.
**Figure S5:** Number of individuals of *Chlorocebus sabaeus*.
**Figure S6:** Number of individuals of *Galago senegalensis*.
**Figure S7:** Number of individuals of *Hystrix cristata*.
**Figure S8:** Number of individuals of *Lepus* sp.
**Figure S9:** Number of individuals of *Lupulella adusta*.
**Figure S10:** Number of individuals of *Lycaon pictus*.
**Figure S11:** Number of individuals of *Aonyx capensis*.
**Figure S12:** Number of individuals of *Mellivora capensis*.
**Figure S13:** Number of individuals of *Panthera leo*.
**Figure S14:** Number of individuals of *Panthera pardus*.
**Figure S15:** Number of individuals of *Leptailurus serval*.
**Figure S16:** Number of individuals of *Felis lybica*.
**Figure S17:** Number of individuals of *Crocuta crocuta*.
**Figure S18:** Number of individuals of *Civettictis civetta*.
**Figure S19:** Number of individuals of *Genetta genetta*.
**Figure S20:** Number of individuals of *Genetta pardina*.
**Figure S21:** Number of individuals of *Atilax paludinosus*.
**Figure S22:** Number of individuals of *Herpestes ichneumon*.
**Figure S23:** Number of individuals of *Herpestes sanguineus*.
**Figure S24:** Number of individuals of *Ichneumia albicauda*.
**Figure S25:** Number of individuals of *Mungos mungo*.
**Figure S26:** Number of individuals of *Mungos gambianus*.
**Figure S27:** Number of individuals of *Phacochoerus africanus*.
**Figure S28:** Number of individuals of *Potamochoerus porcus*.
**Figure S29:** Number of individuals of *Hippopotamus amphibius*.
**Figure S30:** Number of individuals of *Syncerus caffer*.
**Figure S31:** Number of individuals of *Tragelaphus scriptus*.
**Figure S32:** Number of individuals of *Sylvicapra grimmia*.
**Figure S33:** Number of individuals of *Cephalophorus rufilatus*.
**Figure S34:** Number of individuals of *Ourebia ourebi*.
**Figure S35:** Number of individuals of *Kobus kob kob*.
**Figure S36:** Number of individuals of *Kobus ellipsiprymnus defassa*.
**Figure S37:** Number of individuals of *Alcelaphus buselaphus major*.
**Figure S38:** Number of individuals of *Hippotragus equinus koba*.
**Table S1:** Model results on species count, animal sighting rates, and evenness including the interaction between habitat and season as predictor; estimates, standard errors, confidence intervals, likelihood ratio tests, significance tests, and range of estimates derived from dropping each sampling site one at a time.
**Table S2:** Relative sighting frequencies across three habitat types during each season for medium to large mammal species with at least 25 total records from February 2022 to March 2023. Gradual shading of cells reflects the proportion of sightings per habitat and season (white = 0, dark green = 1), providing a visual guide alongside the printed values.
**Table S3:** Overview of the fitted species‐specific models. We fitted species‐specific models for species for which at least one record was available for every habitat–season combination.
**Table S4:** Model results on species‐specific sighting frequencies in relation to the interaction between habitat and season; estimates, standard errors, confidence intervals, likelihood ratio tests, significance tests, and range of estimates derived from dropping each sampling site one at a time. To account for multiple testing, *p*‐values were adjusted using the Benjamini–Hochberg false discovery rate correction.

## Data Availability

All data and scripts are available on OSF (https://doi.org/10.17605/OSF.IO/RG9A7).

## References

[ece374005-bib-0001] Anderson, T. M. , S. White , B. Davis , et al. 2016. “The Spatial Distribution of African Savannah Herbivores: Species Associations and Habitat Occupancy in a Landscape Context.” Philosophical Transactions of the Royal Society of London. Series B, Biological Sciences 371, no. 1703: 20150314. 10.1098/rstb.2015.0314. 27502379 PMC4978872

[ece374005-bib-0002] Arbonnier, M. 2002. Arbres, Arbustes et Lianes des Zones Sèches d'Afrique de l'Ouest. Vol. 2. CIRAD, MNHN.

[ece374005-bib-0003] Bauer, H. , B. Chardonnet , P. Scholte , et al. 2021. “Consider Divergent Regional Perspectives to Enhance Wildlife Conservation Across Africa.” Nature Ecology & Evolution 5, no. 2: 149–152. 10.1038/s41559-021-01491-3. 33139922

[ece374005-bib-0004] Bolker, B. , and others . 2025. “GLMM FAQ.” Accessed June 5, 2026. https://bbolker.github.io/mixedmodels‐misc/glmmFAQ.html#underdispersion.

[ece374005-bib-0005] Bond, W. J. 2019. Open Ecosystems: Ecology and Evolution Beyond the Forest Edge. Oxford University Press.

[ece374005-bib-0006] Bonnot, N. , N. Morellet , H. Verheyden , et al. 2013. “Habitat Use Under Predation Risk: Hunting, Roads and Human Dwellings Influence the Spatial Behaviour of Roe Deer.” European Journal of Wildlife Research 59: 185–193. 10.1007/s10344-012-0665-8.

[ece374005-bib-0007] Boyers, M. , F. Parrini , N. Owen‐Smith , B. F. N. Erasmus , and R. S. Hetem . 2019. “How Free‐Ranging Ungulates With Differing Water Dependencies Cope With Seasonal Variation in Temperature and Aridity.” Conservation Physiology 7, no. 1: coz064. 10.1093/conphys/coz064. 31723430 PMC6839429

[ece374005-bib-0008] Brain, C. , and D. Mitchell . 1999. “Body Temperature Changes in Free‐Ranging Baboons (* Papio hamadryas Ursinus*) in the Namib Desert, Namibia.” International Journal of Primatology 20, no. 4: 585–598. 10.1023/A:1020394824547.

[ece374005-bib-0009] Brooks, M. E. , K. Kristensen , K. J. Benthem , et al. 2017. “glmmTMB Balances Speed and Flexibility Among Packages for Zero‐Inflated Generalized Linear Mixed Modeling.” R Journal 9, no. 2: 378. 10.32614/RJ-2017-066.

[ece374005-bib-0010] Buisson, E. , S. Le Stradic , F. A. O. Silveira , et al. 2019. “Resilience and Restoration of Tropical and Subtropical Grasslands, Savannas, and Grassy Woodlands.” Biological Reviews 94: 590–609. 10.1111/brv.12470. 30251329

[ece374005-bib-0011] Burgess, N. , J. Hales , E. Underwood , et al. 2004. Terrestrial Eco‐Regions of Africa and Madagascar: A Conservation Assessment. World Wildlife Fund.

[ece374005-bib-0012] Burkepile, D. E. , C. E. Burns , C. J. Tambling , et al. 2013. “Habitat Selection by Large Herbivores in a Southern African Savanna: The Relative Roles of Bottom‐Up and Top‐Down Forces.” Ecosphere 4, no. 11: 1–19. 10.1890/ES13-00078.1.

[ece374005-bib-0013] Casaer, J. , T. Milotic , Y. Liefting , P. Desmet , and P. Jansen . 2019. “Agouti: A Platform for Processing and Archiving of Camera Trap Images.” Biodiversity Information Science and Standards 3: e46690. 10.3897/biss.3.46690.

[ece374005-bib-0014] Craigie, I. D. , J. E. Baillie , A. Balmford , et al. 2010. “Large Mammal Population Declines in Africa's Protected Areas.” Biological Conservation 143, no. 9: 2221–2228. 10.1016/j.biocon.2010.06.007.

[ece374005-bib-0015] Cusack, J. J. , A. J. Dickman , M. Kalyahe , et al. 2017. “Revealing Kleptoparasitic and Predatory Tendencies in an African Mammal Community Using Camera Traps: A Comparison of Spatiotemporal Approaches.” Oikos 126, no. 6: 812–822. 10.1111/oik.03403.

[ece374005-bib-0016] Dobson, A. J. 2002. An Introduction to Generalized Linear Models. Chapman & Hall/CRC.

[ece374005-bib-0017] Dudley, N. , L. Eufemia , M. Fleckenstein , M. E. Periago , I. Petersen , and J. F. Timmers . 2020. “Grasslands and Savannahs in the UN Decade on Ecosystem Restoration.” Restoration Ecology 28, no. 6: 1313–1317. 10.1111/rec.13272.

[ece374005-bib-0018] Dupuy, A. R. 1971. Le Niokolo‐Koba, Premier Grand Parc National de la République du Sénégal. GIA.

[ece374005-bib-0019] Dzingwena, L. , L. Thel , M. Choisy , et al. 2025. “Climate and Predation Drive Variation of Diel Activity Patterns in Chacma Baboons ( *Papio ursinus* ) Across Southern Africa.” Scientific Reports 15, no. 1: 39342. 10.1038/s41598-025-23151-3. 41214118 PMC12603080

[ece374005-bib-0020] Fagen, R. M. , and R. N. Goldman . 1977. “Behavioural Catalogue Analysis Methods.” Animal Behaviour 25: 261–274. 10.1016/0003-3472(77)90001-X.

[ece374005-bib-0021] Fischer, J. , G. H. Kopp , F. Dal Pesco , et al. 2017. “Charting the Neglected West: The Social System of Guinea Baboons.” American Journal of Physical Anthropology 162: 15–31. 10.1002/ajpa.23144. 28105722 PMC6586040

[ece374005-bib-0061] Frey, S. , J. T. Fisher , A. C. Burton , and J. P. Volpe . 2017. “Investigating Animal Activity Patterns and Temporal Niche Partitioning Using Camera‐Trap Data: Challenges and Opportunities.” Remote Sensing in Ecology and Conservation 3, no. 3: 123–132. 10.1002/rse2.60.

[ece374005-bib-0022] Gaynor, K. M. , C. E. Hojnowski , N. H. Carter , and J. S. Brashares . 2018. “The Influence of Human Disturbance on Wildlife Nocturnality.” Science 360, no. 6394: 1232–1235. 10.1126/science.aar7121. 29903973

[ece374005-bib-0060] Grabowski, K. L. , E. M. Phillips , and K. M. Gaynor . 2024. “Limited Spatiotemporal Niche Partitioning Among Mesocarnivores in Gorongosa National Park, Mozambique.” Ecology and Evolution 14, no. 2: e10965. 10.1002/ece3.10965. 38371865 PMC10869889

[ece374005-bib-0023] Gueye, M. , R. Pellaton , D. Van Cauteren , et al. 2024. “Population Size and Social Structure of Lions in a West African Protected Area.” African Journal of Ecology 62, no. 1: e13226. 10.1111/aje.13226.

[ece374005-bib-0024] Havmøller, R. W. , N. S. Jacobsen , N. Scharff , F. Rovero , and F. Zimmermann . 2020. “Assessing the Activity Pattern Overlap Among Leopards ( *Panthera pardus* ), Potential Prey and Competitors in a Complex Landscape in Tanzania.” Journal of Zoology 311, no. 3: 175–182. 10.1111/jzo.12774.

[ece374005-bib-0025] Hayward, M. W. , and R. Slotow . 2009. “Temporal Partitioning of Activity in Large African Carnivores: Tests of Multiple Hypotheses.” South African Journal of Wildlife Research 39, no. 2: 109–125.

[ece374005-bib-0026] Horion, R. , Z. Woodgate , and M. Drouilly . 2024. “First Insights Into the Spatio‐Temporal Ecology of Sympatric Large Carnivores in Niokolo‐Koba National Park, Senegal.” Oryx 58, no. 5: 664–675. 10.1017/S0030605323001746.

[ece374005-bib-0027] Houéhounha, D. H. M. , S. Lhoest , J. Ohouko , et al. 2025. “African Conservation Success: Niokolo‐Koba National Park (Senegal) Removed From the List of World Heritage in Danger.” Heritage 8, no. 10: 403. 10.3390/heritage8100403.

[ece374005-bib-0028] IUCN . 2026. “The IUCN Red List of Threatened Species.” Version 2025‐2. Accessed Jan 13, 2026. https://www.iucnredlist.org.

[ece374005-bib-0029] Kane, M. D. 2014. “Estimating Abundance, Density, and Occupancy of Lion, Leopard and Serval in the Niokolo Koba National Park in Senegal.” PhD Thesis, Virginia Polytechnic Institute and State University.

[ece374005-bib-0030] Kingdon, J. , D. Happold , T. Butynski , M. Hoffmann , M. Happold , and J. Kalina , eds. 2013. Mammals of Africa (6 Vols). Bloomsbury Publishing.

[ece374005-bib-0031] Klapproth, M. 2010. “Classification of the Guinea Baboon Habitat at Simenti (Niokolo Koba National Park, Senegal) by Means of Remote Sensing.” MSc Thesis. Georg‐August‐University Göttingen.

[ece374005-bib-0032] Kuhner, M. , K. Gobush , Z. Kaliszewska , R. Horwitz , and S. Wasser . 2025. “Distribution of African Savanna Elephants ( *Loxodonta africana* ), African Forest Elephants ( *L. cyclotis* ), and Their Hybrids Across Africa Based on Genetic Evidence.” Global Ecology and Conservation 59: e03530. 10.1016/j.gecco.2025.e03530.

[ece374005-bib-0033] Lindshield, S. , S. L. Bogart , M. Gueye , P. I. Ndiaye , and J. D. Pruetz . 2019. “Informing Protection Efforts for Critically Endangered Chimpanzees ( *Pan troglodytes verus* ) and Sympatric Mammals Amidst Rapid Growth of Extractive Industries in Senegal.” Folia Primatologica 90, no. 2: 124–136. 10.1159/000496145. 30826809

[ece374005-bib-0062] McCullagh, P. , and J. A. Nelder . 1989. Generalized Linear Models. 2nd ed. Chapman & Hall.

[ece374005-bib-0034] Meredith, M. , M. Ridout , and L. A. D. Campbell . 2024. “Package ‘overlap’.” R Package Version 0.3.9.

[ece374005-bib-0035] Mirghani, N. , M. Llana , A. Barciela , et al. 2025. “Uncovering Cryptic Diversity: Camera Trap Insights Into the Effects of Seasonality and Anthropogenic Presence in a Mosaic Savannah Ecosystem.” African Journal of Ecology 63, no. 4: e70069. 10.1111/aje.70069.

[ece374005-bib-0036] Nakazawa, N. 2023. “Overlap of Activity Patterns Between Leopards and Their Potential Prey Species in Mahale Mountains National Park, Tanzania.” Journal of Zoology 319, no. 3: 188–199. 10.1111/jzo.13037.

[ece374005-bib-0037] Ndiaye, M. M. , M. Drouilly , A. A. Senghor , et al. 2024. “Rediscovery of the Endangered Giant Pangolin (*Smutsia gigantea*) in Senegal After 24 Years.” African Journal of Ecology 62, no. 2: e13279. 10.1111/aje.13279.

[ece374005-bib-0038] Ohrndorf, L. , R. Mundry , J. Beckmann , J. Fischer , and D. Zinner . 2025a. “Impact of Food Availability and Predator Presence on Patterns of Landscape Partitioning Among Neighbouring Guinea Baboon ( *Papio papio* ) Parties.” Movement Ecology 13, no. 1: 9. 10.1186/s40462-025-00534-9. 39987137 PMC11847332

[ece374005-bib-0039] Ohrndorf, L. , R. Mundry , J. Beckmann , J. Fischer , and D. Zinner . 2025b. “Spatiotemporal Patterns of Sleeping Site Use of Guinea Baboon Parties ( *Papio papio* ).” Ecology and Evolution 15, no. 7: e71610. 10.1002/ece3.71610. 40718691 PMC12296831

[ece374005-bib-0040] Oksanen, J. , G. Simpson , F. Blanchet , et al. 2025. “vegan: Community Ecology Package.” R Package Version 2.8‐0. 10.32614/CRAN.package.vegan.

[ece374005-bib-0041] Panthera . 2025. Last “Ghost Elephant” Spotted on Camera. Panthera Accessed Dec 09, 2025. https://panthera.org/blog‐post/last‐ghost‐elephant‐spotted‐camera.

[ece374005-bib-0042] R Core Team . 2024. R: A Language and Environment for Statistical Computing [Computer Software]. R Foundation for Statistical Computing.

[ece374005-bib-0043] Rabeil, T. , P. Hejcmanová , M. Gueye , R. Greffrath , and D. Cornut . 2018. Inventaire Combiné Terrestre et Aérien, Parc National du Niokolo‐Koba, Sénégal. Direction des Parcs Nationaux du Sénégal.

[ece374005-bib-0045] Scholte, P. , O. Pays , B. Chardonnet , A. Ouattara , and D. Tiomoko . 2025. “Large Mammal Population Trends in Comoé National Park (1958–2022): Towards Understanding Their Asymmetric Decline and Recovery in West Africa's Largest Savanna Park.” PLoS One 20, no. 5: e0320455. 10.1371/journal.pone.0320455. 40435220 PMC12118930

[ece374005-bib-0046] Shorrocks, B. 2007. The Biology of African Savannahs. Oxford University Press.

[ece374005-bib-0047] Stehlíková, B. K. 2023. “Population Structure of Critically Endangered Western Derby Eland (*Tragelaphus derbianus derbianus*) in Niokolo Koba National Park, Senegal.” PhD Thesis, Czech University of Life Sciences Prague.

[ece374005-bib-0048] Terrien, J. , M. Perret , and F. Aujard . 2011. “Behavioral Thermoregulation in Mammals: A Review.” Frontiers in Bioscience 16, no. 4: 1428–1444. 10.2741/3797. 21196240

[ece374005-bib-0049] Thieurmel, B. , A. Elmarhraoui , and M. B. Thieurmel . 2019. Package ‘suncalc’.” R Package Version 0.5.

[ece374005-bib-0050] UNESCO World Heritage Centre . 2025. “Niokolo‐Koba National Park.” Accessed Jan 13, 2026. https://whc.unesco.org/en/list/153/.

[ece374005-bib-0051] UNESCO World Heritage Committee . 1981. “5 COM VIII.15. Nominations to the World Heritage List (Inscribed Sites).” Accessed Jan 13, 2026. https://whc.unesco.org/en/decisions/5236.

[ece374005-bib-0052] UNESCO World Heritage Committee . 2007. “Decision 31 COM 8C.1. Inscriptions on the World Heritage List in Danger.” Accessed August 14, 2025. https://whc.unesco.org/en/decisions/1251.

[ece374005-bib-0053] UNESCO World Heritage Committee . 2024. “Decision 46 COM 8C.3. Update of the List of World Heritage in Danger (Removed Properties).” Accessed August 14, 2025. https://whc.unesco.org/en/decisions/8639.

[ece374005-bib-0054] UNESCO World Heritage Committee . 2025. “47 COM 7B.50. Niokolo Koba National Park (Senegal).” Accessed Jan 15, 2026. https://whc.unesco.org/en/decisions/8774.

[ece374005-bib-0055] van der Hoek, Y. , E. Binyinyi , U. Ngobobo , T. S. Stoinski , and D. Caillaud . 2023. “Diversity and Diel Activity Patterns of Terrestrial Mammals in the Nkuba Conservation Area, Democratic Republic of the Congo.” Oryx 57, no. 1: 107–117. 10.1017/S003060532100106X.

[ece374005-bib-0056] Vazquez, C. , J. M. Rowcliffe , K. Spoelstra , and P. A. Jansen . 2019. “Comparing Diel Activity Patterns of Wildlife Across Latitudes and Seasons: Time Transformations Using Day Length.” Methods in Ecology and Evolution 10, no. 12: 2057–2066. 10.1111/2041-210X.13290.

[ece374005-bib-0057] Versavaud, L. , P. Aczel , U. Nkounkou , C. Portal , J. F. Gerard , and N. Giotto . 2026. “Effects of Phylogeny, Stratum and Season on the Diel Activity Patterns of Mammals in a Lowland Rainforest of the Republic of Congo.” African Journal of Ecology 64, no. 1: e70142. 10.1111/aje.70142.

[ece374005-bib-0058] Wessling, E. G. , P. Dieguez , M. Llana , L. Pacheco , J. D. Pruetz , and H. S. Kühl . 2020. “Chimpanzee ( *Pan troglodytes verus* ) Density and Environmental Gradients at Their Biogeographical Range Edge.” International Journal of Primatology 41, no. 6: 822–848. 10.1007/s10764-020-00182-3.

[ece374005-bib-0059] Zinner, D. , M. Klapproth , A. Schell , et al. 2021. “Comparative Ecology of Guinea Baboons ( *Papio papio* ).” Primate Biology 8, no. 1: 19–35. 10.5194/pb-8-19-2021. 34109265 PMC8182668

