# Supplementary material for: Diversity and Spatiotemporal Activity Patterns of Medium and Large Mammals in the Niokolo‐Koba National Park, Senegal
Source: Ecol Evol. 2026 Jul 10;16(7):e74005. doi: 10.1002/ece3.74005 (PMC13354549; doi:10.1002/ece3.74005)
Supplement: Supplementary file 1 — Figure S1: Relationship between pairwise spatial distance and pairwise residual difference for (a) species count, (b) number of sightings, and (c) evenness. Each point represents a unique pair of sampling sites (n = 666). Spatial autocorrelation would result in a clear clustering of residual differences with spatial distance. Pearson correlation coefficients were low (species count: r = 0.045; sightings: r = −0.010; evenness: r = 0.030), suggesting little to no spatial autocorrelation. Figure S2: Number of individuals of Orycteropus afer. Figure S3: Number of individuals of Papio papio. Figure S4: Number of individuals of Erythrocebus patas. Figure S5: Number of individuals of Chlorocebus sabaeus. Figure S6: Number of individuals of Galago senegalensis. Figure S7: Number of individuals of Hystrix cristata. Figure S8: Number of individuals of Lepus sp. Figure S9: Number of individuals of Lupulella adusta. Figure S10: Number of individuals of Lycaon pictus. Figure S11: Number of individuals of Aonyx capensis. Figure S12: Number of individuals of Mellivora capensis. Figure S13: Number of individuals of Panthera leo. Figure S14: Number of individuals of Panthera pardus. Figure S15: Number of individuals of Leptailurus serval. Figure S16: Number of individuals of Felis lybica. Figure S17: Number of individuals of Crocuta crocuta. Figure S18: Number of individuals of Civettictis civetta. Figure S19: Number of individuals of Genetta genetta. Figure S20: Number of individuals of Genetta pardina. Figure S21: Number of individuals of Atilax paludinosus. Figure S22: Number of individuals of Herpestes ichneumon. Figure S23: Number of individuals of Herpestes sanguineus. Figure S24: Number of individuals of Ichneumia albicauda. Figure S25: Number of individuals of Mungos mungo. Figure S26: Number of individuals of Mungos gambianus. Figure S27: Number of individuals of Phacochoerus africanus. Figure S28: Number of individuals of Potamochoerus porcus. Figure S29: Number of individ [file ECE3-16-e74005-s001.pdf]

Supplementary material

Diversity and spatiotemporal activity patterns of medium and large mammals in the Niokolo Koba National Park, Senegal

Lisa Ohrndorf[1,2], Augustin Brouillet[1,2], Annika M. Zuleger[3,4], Ndiouga Diakhaté[1,2], Djibril Coly[5], Chérif Younousse Kéba Camara[5], Amadou Bamba Diedhiou[5], Irene Gutiérrez Díez[6], Julia Fischer[1,2], Dietmar Zinner[1,2]

[1] Georg-August-Universität Göttingen, Johann-Friedrich-Blumenbach Institute, Department for Primate Cognition, Göttingen, Germany

[2] German Primate Center - Leibniz Institute for Primate Research, Cognitive Ethology Laboratory, Göttingen, Germany

[3] Institute of Biology, Martin Luther University Halle-Wittenberg, Halle (Saale), Germany

[4] German Centre for Integrative Biodiversity Research (iDiv) Halle-Jena-Leipzig, Leipzig, Germany

[5] Direction Des Parcs Nationaux (DPN), Dakar, Senegal

[6]Centro de cría del lince ibérico Zarza de Granadilla, Tragsatec. Cáceres, Spain

Table S1: Model results on species count, animal sighting rates, and evenness including the interaction between habitat and season as predictor; estimates, standard errors, confidence intervals, likelihood ratio tests, significance tests, and range of estimates derived from dropping each sampling site one at a time.

Model	Term	Estimate	SE	CI_{Lower}	CI_{Upper}	$\chi 2$	df	P	Min	Max
Species count	Intercept	-2.686	0.121	-2.937	-2.459				-2.726	-2.615
	habitat.savannah[a]	-0.010	0.143	-0.378	0.120				-0.169	-0.061
	habitat.wetland[a]	-0.214	0.305	-0.855	0.359				-0.590	0.087
	season.wet[b]	0.024	0.130	-0.232	0.285				-0.010	0.087
	habitat.savannah[a]: season.wet[b]	0.262	0.153	-0.043	0.572	2.991	2	0.224	0.199	0.296
	habitat.wetland[a]: season.wet[b]	0.137	0.333	-0.610	0.748				0.055	0.275
Sighting rate	Intercept	0.079	0.391	-0.699	0.766				-0.083	0.438
	habitat.savannah[a]	-0.605	0.460	-1.488	0.259				-0.953	-0.437
	habitat.wetland[a]	-0.281	0.949	-2.316	1.510				-0.871	0.201
	season.wet[b]	-0.719	0.326	-1.350	-0.111				-0.925	-0.526
	habitat.savannah[a]: season.wet[b]	0.684	0.385	-0.045	1.411	3.042	2	0.218	0.493	0.892
	habitat.wetland[a]: season.wet[b]	0.665	0.839	-0.877	2.208				-1.132	1.636
Evenness	Intercept	-5.216	0.141	-5.502	-4.933				-5.309	-5.143
	habitat.savannah[a]	0.467	0.168	0.139	0.847				0.394	0.560
	habitat.wetland[a]	0.147	0.344	-0.460	0.802				-0.327	0.637
	season.wet[b]	0.365	0.162	0.018	0.684				0.284	0.447
	habitat.savannah[a]: season.wet[b]	0.231	0.195	-0.144	0.614	2.177	2	0.337	0.150	0.312
	habitat.wetland[a]: season.wet[b]	0.513	0.409	-0.207	1.317				0.086	0.920

a = variable dummy coded with the reference level being forest. The indicated test refers to the overall effect of habitat derived from the full-null model comparisons.
b = variable dummy coded with the reference level being dry season. The indicated test refers to the overall effect of season derived from the full-null model comparison.

Table S2: Relative sighting frequencies across three habitat types during each season for medium to large mammal species with at least 25 total records from February 2022 to March 2023. Gradual shading of cells reflects the proportion of sightings per habitat and season (white = 0, dark green = 1), providing a visual guide alongside the printed values.

Species	dry			wet		
	forest	savannah	wetland	forest	savannah	wetland
Orycteropus afer	0.11	0.89	0.00	0.27	0.73	0.00
Papio papio	0.56	0.40	0.04	0.66	0.32	0.02
Erythrocebus patas	0.15	0.83	0.02	0.03	0.97	0.00
Chlorocebus sabaeus	0.72	0.26	0.02	0.75	0.25	0.00
Galago senegalensis	0.06	0.94	0.00	0.13	0.88	0.00
Hystrix cristata	0.67	0.33	0.00	0.64	0.36	0.00
Lupulella adusta	0.00	0.99	0.01	0.00	1.00	0.00
Panthera leo	0.17	0.78	0.06	0.43	0.57	0.00
Panthera pardus	0.88	0.05	0.07	0.67	0.29	0.04
Crocuta crocuta	0.40	0.60	0.00	0.13	0.86	0.02
Civettictis civetta	0.59	0.41	0.00	0.20	0.70	0.10
Genetta genetta	0.00	0.99	0.01	0.00	0.92	0.08
Genetta pardina	0.85	0.06	0.09	0.80	0.05	0.16
Herpestes ichneumon	0.50	0.50	0.00	0.20	0.80	0.00
Ichneumia albicauda	0.39	0.59	0.02	0.32	0.64	0.03
Mungos mungo	0.56	0.44	0.00	0.54	0.46	0.00
Mungos gambianus	0.00	1.00	0.00	0.08	0.92	0.00
Phacochoerus africanus	0.23	0.65	0.12	0.19	0.45	0.36
Potamochoerus porcus	0.25	0.75	0.00	0.13	0.88	0.00
Hippopotamus amphibius	0.79	0.07	0.14	0.71	0.29	0.00
Tragelaphus scriptus	0.46	0.51	0.03	0.40	0.49	0.10
Sylvicapra grimmia	0.01	0.99	0.00	0.00	1.00	0.00
Cephalophorus rufilatus	0.71	0.25	0.05	0.35	0.49	0.16
Ourebia ourebi	0.01	0.99	0.01	0.00	1.00	0.00
Kobus ellipsiprymnus	0.33	0.51	0.16	0.16	0.54	0.30
Kobus kob	0.03	0.39	0.58	0.02	0.86	0.12
Hippotragus equinus	0.11	0.86	0.03	0.01	0.98	0.00

As a supplementary analysis to assess whether individual species differed in their detection frequencies across habitats and seasons, we fitted separate generalised linear mixed models following the same approach as for the models on species count and sighting frequencies (Table S3). Species-specific analyses were restricted to species with at least one observation per habitat:season combination. We modelled detection frequency as a function of habitat, season, and their interaction. We assessed zero-inflation by simulating response data under the fitted model and its assumed error distribution. We then compared the observed number of zeros with the distribution of expected zeros generated from these simulations. None of the models appeared to be zero-inflated. For all models, we assessed stability by dropping each sampling point from the data one at a time and comparing the estimates from models fitted to these subsets to those from the full dataset. Because sample sizes for individual species were generally low and wetland habitats were underrepresented in our data set, some models were only moderately stable, especially regarding the estimated effect sizes for wetland habitats and their combinations. To account for multiple testing across species-specific models, p-values were adjusted using the Benjamini–Hochberg false discovery rate correction. These analyses should be interpreted cautiously and are intended primarily to identify broad patterns rather than provide robust estimates of species-specific differences.

Table S3: Overview of the fitted species-specific models. We fitted species-specific models for species for which at least one record was available for every habitat–season combination.

Model	Sample size	Error structure	Dispersion parameter
C. rufilatus	396	Poisson	1.185
G. pardina	359	Negative binomial	0.227
H. equinus	1175	Negative binomial	0.630
I. albicauda	381	Poisson	1.170
K. ellipsiprymnus	1941	Negative binomial	0.533
K. kob	1607	Negative binomial	0.495
P. pardus	82	Poisson	0.367
P. papio	3302	Negative binomial	0.561
P. africanus	1589	Negative binomial	0.586
T. scriptus	11056	Negative binomial	0.677

Table S4: Model results on species-specific sighting frequencies in relation to the interaction between habitat and season; estimates, standard errors, confidence intervals, likelihood ratio tests, significance tests, and range of estimates derived from dropping each sampling site one at a time. To account for multiple testing, p-values were adjusted using the Benjamini–Hochberg false discovery rate correction.

Model	Term	Estimate	SE	CI_{Lower}	CI_{Upper}	χ2	df	P	Min	Max
C. rufilatus	Intercept	-2.920	0.501	-3.900	-1.968				-3.108	-2.573
	habitat.savannah[a]	-2.502	0.614	-3.735	-1.356				-2.784	-2.324
	habitat.wetland[a]	-1.722	1.311	-22.038	0.492				-21.343	0.252
	season.wet[b]	-1.283	0.181	-1.655	-0.942				-1.561	-1.039
	habitat.savannah[a]: season.wet[b]	1.334	0.259	0.823	1.883	35.873	2	**<0.001**	0.506	1.612
	habitat.wetland[a]: season.wet[b]	1.707	0.398	-16.995	20.241				-2.542	1.985
G. pardina	Intercept	-5.779	1.571	-11.320	-3.626				-6.622	-5.149
	habitat.savannah[a]	-3.335	1.579	-6.188	0.014				-3.734	-2.483
	habitat.wetland[a]	0.264	3.215	-37.804	6.903				-20.518	3.461
	season.wet[b]	-1.670	0.192	-3.153	-0.986				-1.721	-1.555
	habitat.savannah[a]: season.wet[b]	-0.699	1.044	-26.451	1.479	3.190	2	0.812	-24.322	-0.233
	habitat.wetland[a]: season.wet[b]	0.764	0.463	-18.989	17.947				-1.976	0.815
H. equinus	Intercept	-3.696	0.466	-4.631	-2.959				-4.079	-3.512
	habitat.savannah[a]	1.452	0.511	0.595	2.555				1.270	1.818
	habitat.wetland[a]	0.645	0.996	-1.861	2.313				-0.063	1.208
	season.wet[b]	-2.127	0.671	-4.177	-0.931				-2.343	-1.709
	habitat.savannah[a]: season.wet[b]	2.055	0.742	0.730	4.289	9.393	2	0.073	1.626	2.270
	habitat.wetland[a]: season.wet[b]	0.104	1.430	-19.588	3.969				-16.379	0.431
I. albicauda	Intercept	-4.692	0.908	-6.876	-3.085				-5.220	-4.309
	habitat.savannah[a]	-1.244	1.086	-3.400	0.955				-1.564	-0.709
	habitat.wetland[a]	-1.371	2.294	21.961	2.502				-20.173	0.785
	season.wet[b]	-1.373	0.252	-2.105	-0.812				-2.136	-0.771
	habitat.savannah[a]: season.wet[b]	0.285	0.309	-0.427	1.100	1.633	2	0.875	-0.317	1.048
	habitat.wetland[a]: season.wet[b]	0.972	0.874	-17.659	20.337				-2.161	1.736
K. ellipsi-prymnus	Intercept	-2.199	0.454	-3.095	-1.326				-2.392	-1.930
	habitat.savannah[a]	-0.590	0.535	-1.651	0.455				-0.854	-0.387
	habitat.wetland[a]	0.391	1.085	-2.170	2.331				-1.169	1.944
	season.wet[b]	-0.692	0.444	-1.515	0.203				-0.967	-0.491
	habitat.savannah[a]: season.wet[b]	1.051	0.522	0.035	2.009	4.105	2	0.765	0.846	1.322
	habitat.wetland[a]: season.wet[b]	1.248	1.025	-1.024	3.406				-18.429	1.786

Model	Term	Estimate	SE	CI $_{Lower}$	CI $_{Upper}$	$\chi 2$	df	P	Min	Max
K. kob	Intercept	-5.197	0.763	-7.064	-3.886				-5.680	-4.928
	habitat.savannah[a]	1.639	0.869	0.174	3.853				1.382	2.135
	habitat.wetland[a]	3.845	1.659	0.393	7.102				0.952	6.383
	season.wet[b]	-0.290	0.677	-2.032	1.109				-0.635	0.141
	habitat.savannah[a]: season.wet[b]	0.675	0.757	-0.883	2.392	4.451	2	0.756	0.243	1.019
	habitat.wetland[a]: season.wet[b]	-1.714	1.232	-20.467	1.214				-18.157	-1.376
P. pardus	Intercept	-4.563	0.543	-5.767	-3.546				-4.824	-4.285
	habitat.savannah[a]	-4.074	0.910	-24.421	-2.393				-4.562	-3.736
	habitat.wetland[a]	-0.372	1.283	-21.399	1.872				-0.643	-0.113
	season.wet[b]	-0.808	0.294	-1.570	-0.237				-0.979	-0.568
	habitat.savannah[a]: season.wet[b]	2.197	0.750	0.371	22.412	9.670	2	0.072	1.748	2.604
	habitat.wetland[a]: season.wet[b]	-0.044	1.156	-19.611	2.292				-18.052	0.665
P. papio	Intercept	-0.965	0.372	-1.675	-0.262				-1.172	-0.728
	habitat.savannah[a]	-1.361	0.442	-2.205	-0.517				-1.590	-1.146
	habitat.wetland[a]	-0.909	0.909	-2.704	0.889				-1.486	-0.332
	season.wet[b]	-0.461	0.265	-1.011	0.056				-0.548	-0.307
	habitat.savannah[a]: season.wet[b]	0.132	0.324	-0.520	0.786	0.267	2	0.875	-0.013	0.221
	habitat.wetland[a]: season.wet[b]	-0.113	0.660	-1.820	1.245				-0.369	0.040
P. africanus	Intercept	-2.438	0.420	-3.356	-1.683				-2.674	-2.251
	habitat.savannah[a]	-0.543	0.500	-1.567	0.516				-0.725	-0.319
	habitat.wetland[a]	0.100	1.008	-1.196	2.974				0.664	1.295
	season.wet[b]	-0.291	0.321	-0.929	0.337				-0.476	-0.071
	habitat.savannah[a]: season.wet[b]	0.206	0.383	-0.577	0.948	3.755	2	0.765	-0.012	0.391
	habitat.wetland[a]: season.wet[b]	1.415	0.701	-0.066	2.771				0.695	1.999
T. scriptus	Intercept	0.488	0.322	-0.214	1.128				0.393	0.643
	habitat.savannah[a]	-1.154	0.386	-1.948	-0.364				-1.297	-1.047
	habitat.wetland[a]	-1.010	0.784	-2.800	0.564				-1.428	-0.632
	season.wet[b]	-0.820	0.329	-1.494	-0.099				-1.004	-0.701
	habitat.savannah[a]: season.wet[b]	0.505	0.397	-0.317	1.330	2.323	2	0.875	0.392	0.706
	habitat.wetland[a]: season.wet[b]	1.037	0.840	-0.608	2.729				-0.783	1.851

a = variable dummy coded with the reference level being forest. The indicated test refers to the overall effect of habitat derived from the full-null model comparisons.
b = variable dummy coded with the reference level being dry season. The indicated test refers to the overall effect of season derived from the full-null model comparison.

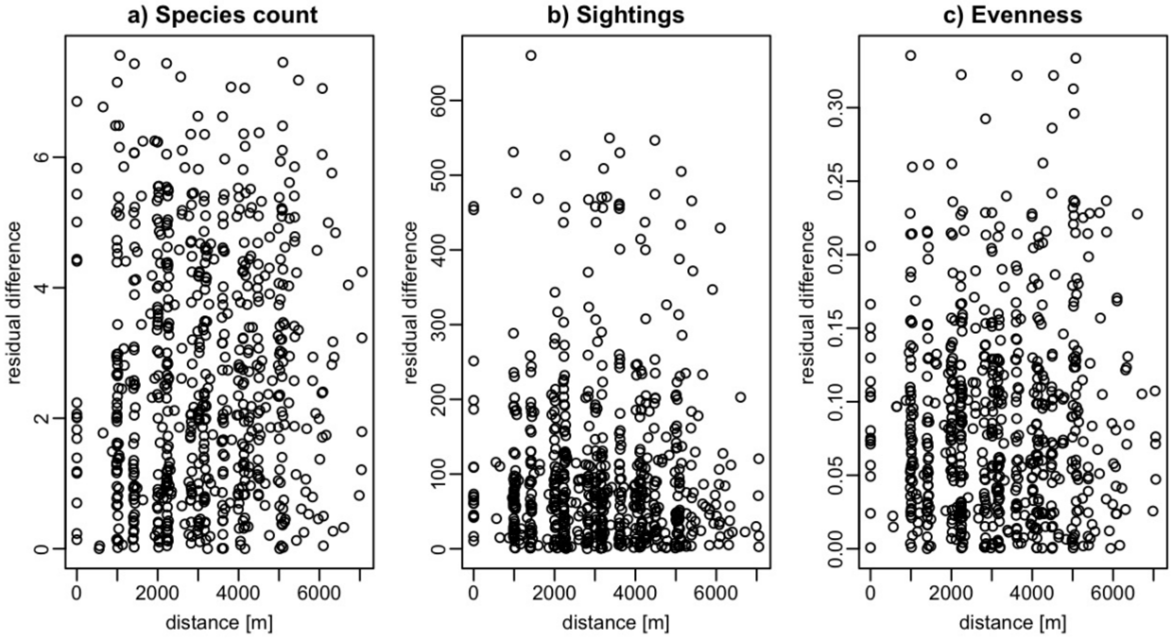

Figure S1: Relationship between pairwise spatial distance and pairwise residual difference for a) species count, b) number of sightings, and c) evenness. Each point represents a unique pair of sampling sites (n = 666). Spatial autocorrelation would result in a clear clustering of residual differences with spatial distance. Pearson correlation coefficients were low (species count: $r = 0.045$; sightings: $r = -0.010$; evenness: $r = 0.030$), suggesting little to no spatial autocorrelation.

In the following Figures S2 to S38, the number of individuals detected per species per sampling site within 100 days of camera trapping across the study site from February 2022 to March 2023 are given.

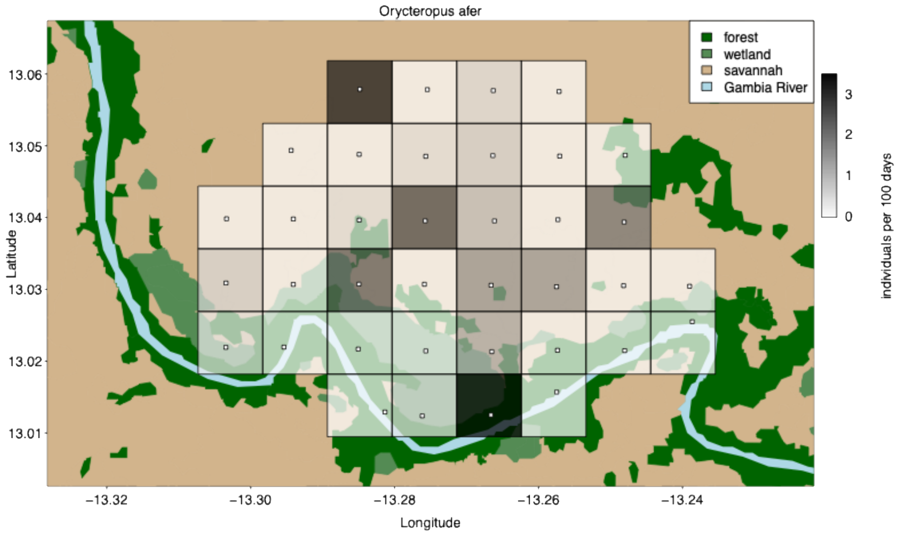

Figure S2**:** Number of individuals of *Orycteropus afer*.

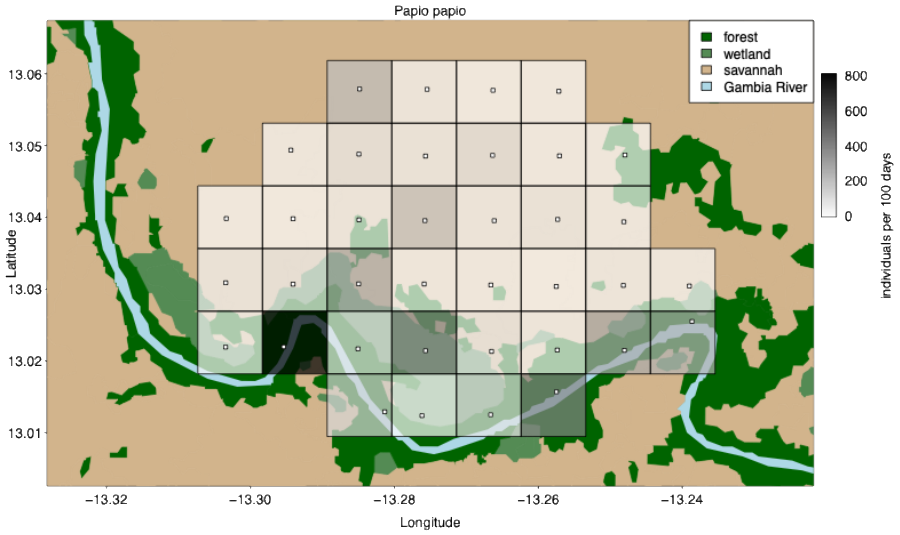

Figure S3: Number of individuals of *Papio papio*.

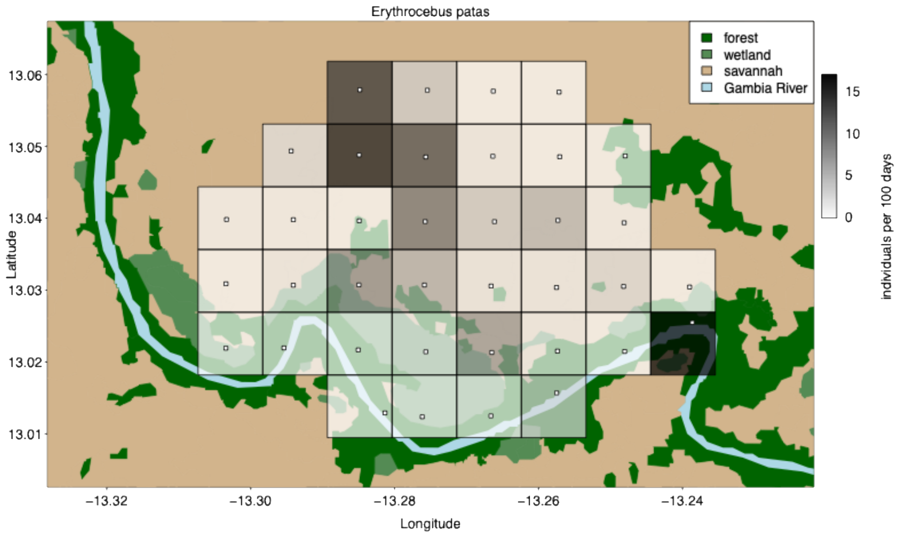

Figure S4: Number of individuals of *Erythrocebus patas*.

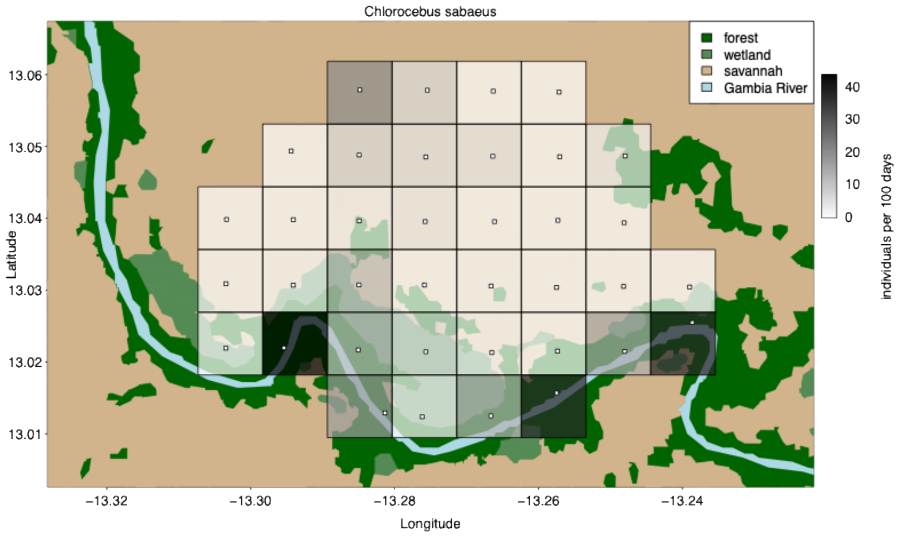

Figure S5: Number of individuals of *Chlorocebus sabaeus*.

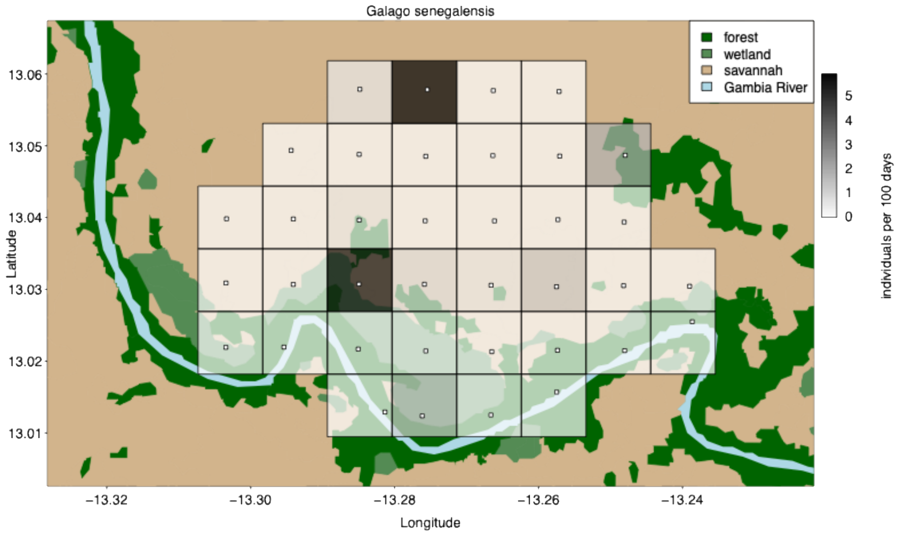

Figure S6: Number of individuals of *Galago senegalensis*.

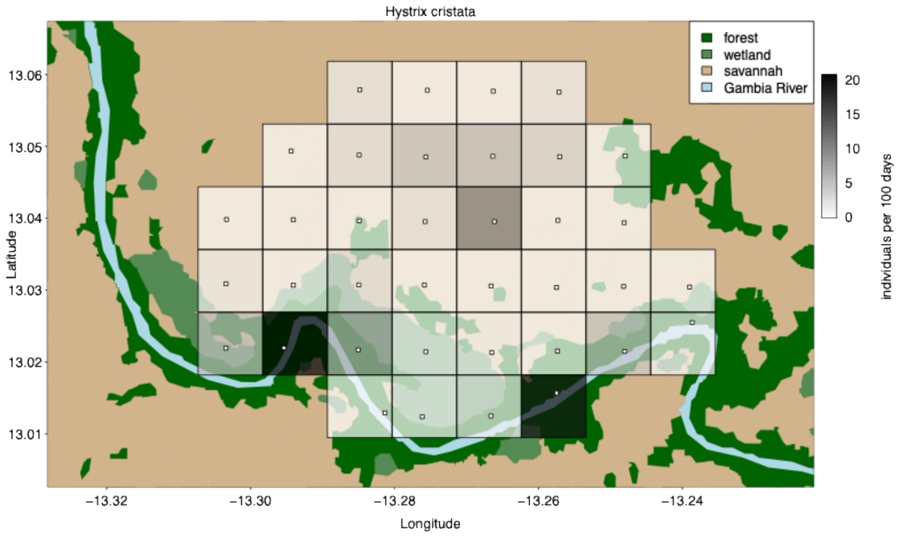

Figure S7: Number of individuals of *Hystrix cristata*.

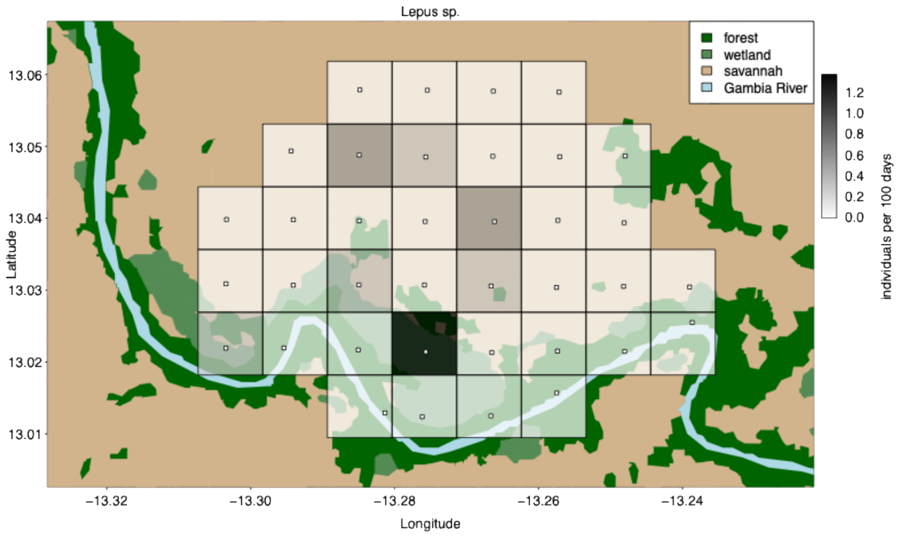

Figure S8: Number of individuals of *Lepus* sp..

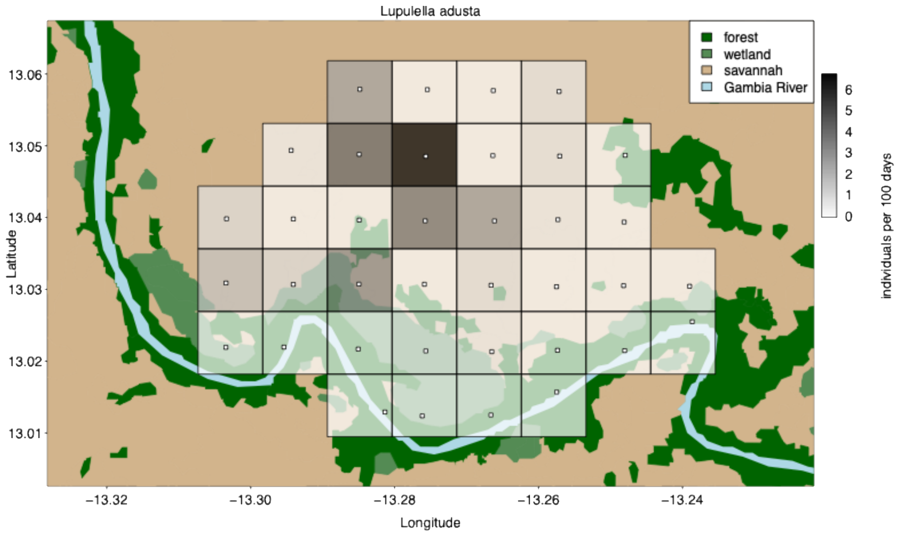

Figure S9: Number of individuals of *Lupulella adusta*.

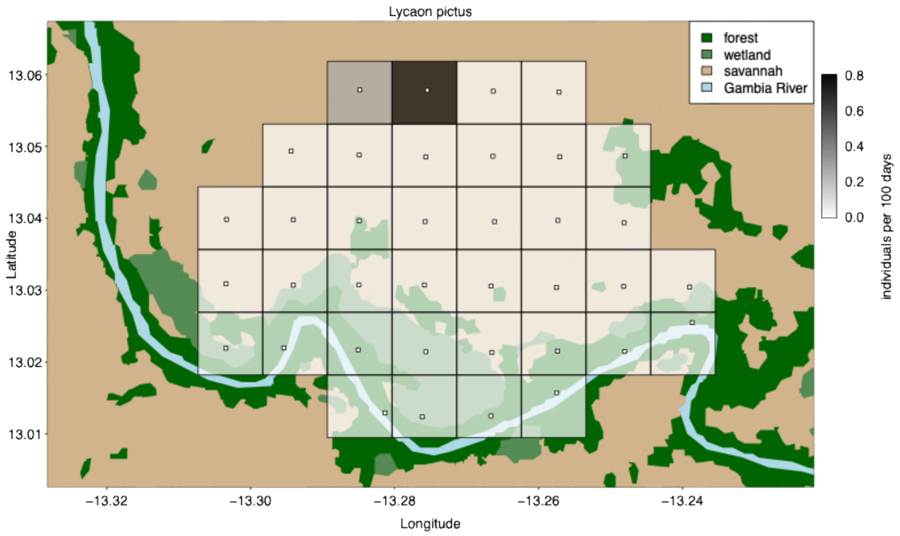

Figure S10: Number of individuals of *Lycaon pictus*.

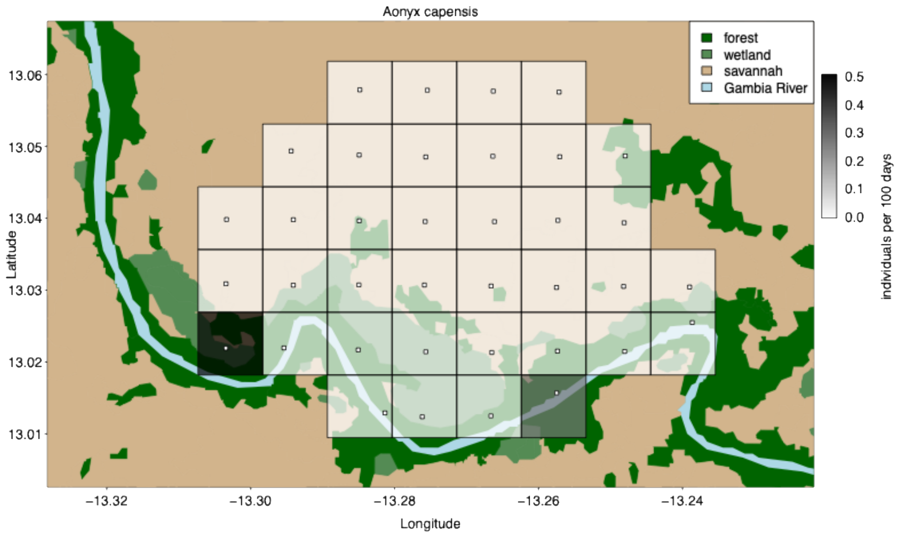

Figure S11: Number of individuals of *Aonyx capensis*.

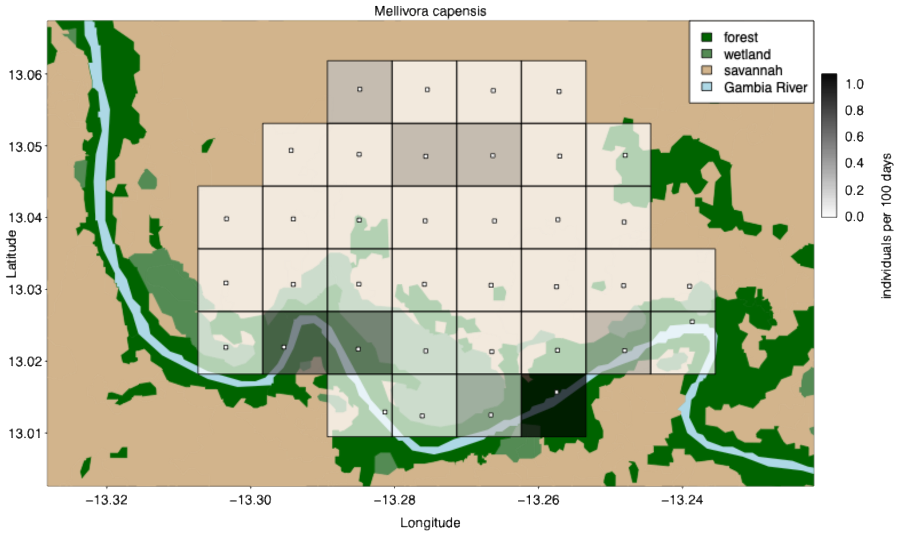

Figure S12: Number of individuals of *Mellivora capensis*.

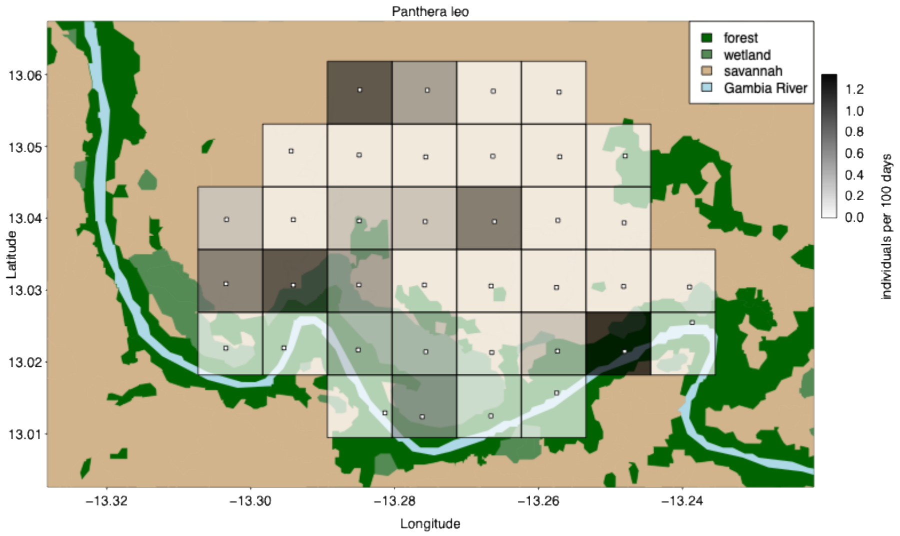

Figure S13: Number of individuals of *Panthera leo*.

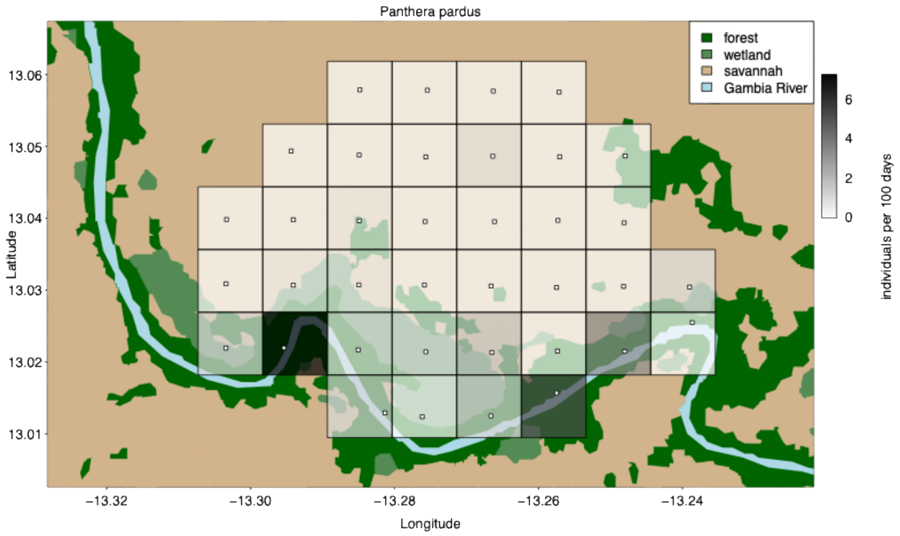

Figure S14: Number of individuals of *Panthera pardus*.

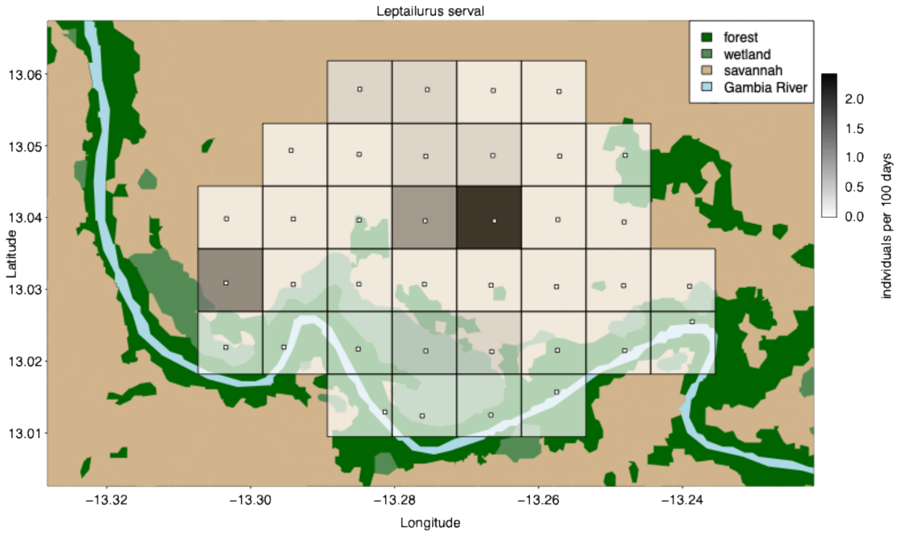

Figure S15: Number of individuals of *Leptailurus serval*.

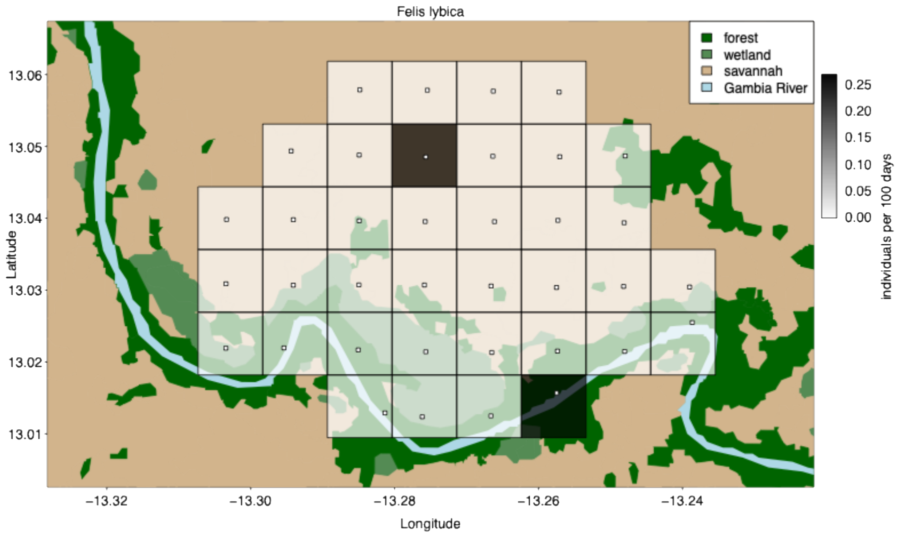

Figure S16: Number of individuals of *Felis lybica*.

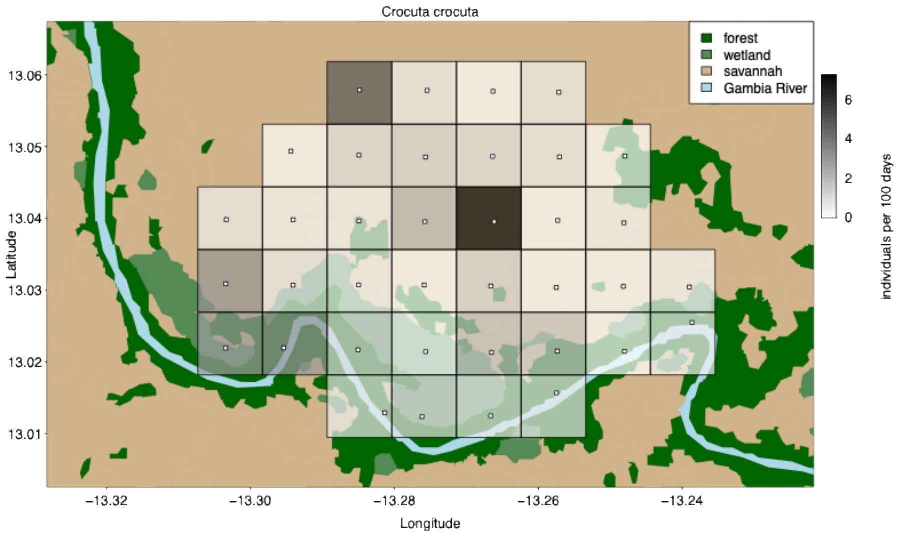

Figure S17: Number of individuals of *Crocuta crocuta*.

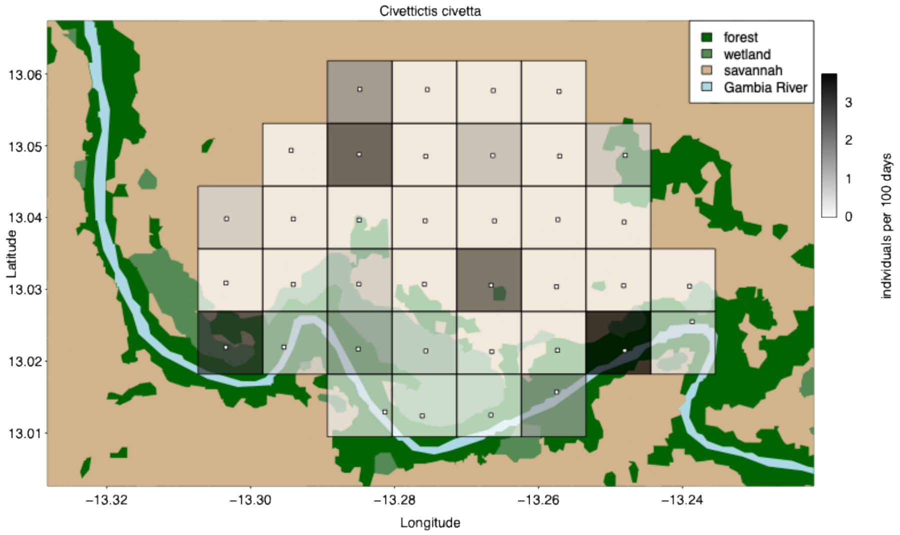

Figure S18: Number of individuals of *Civettictis civetta*.

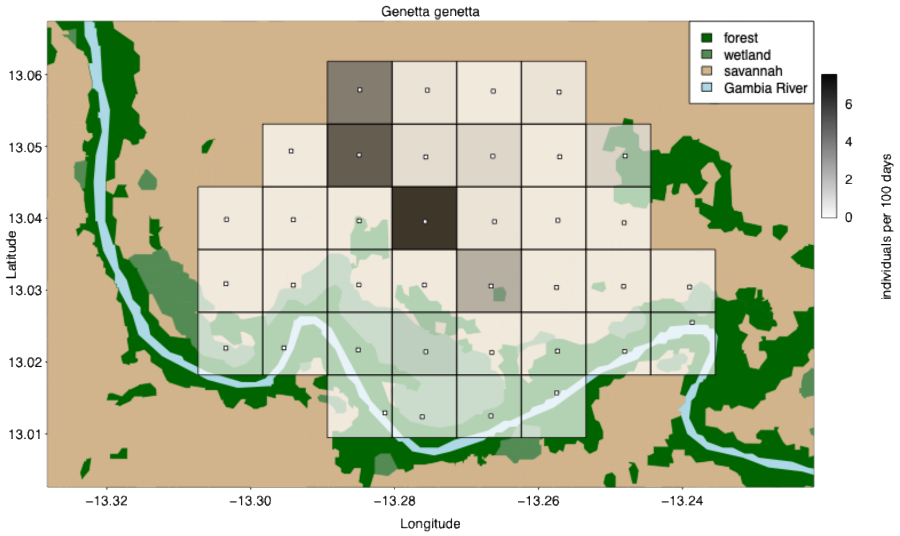

Figure S19: Number of individuals of *Genetta genetta*.

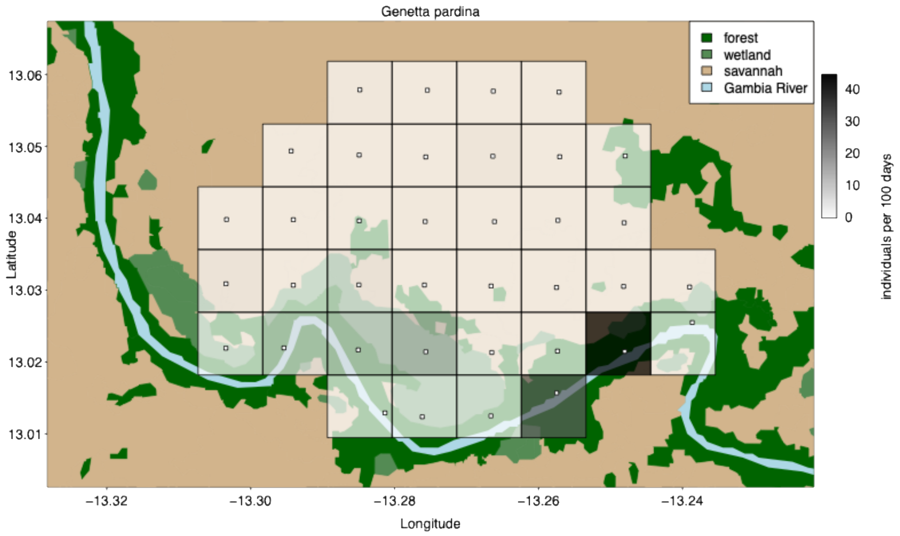

Figure S20: Number of individuals of *Genetta pardina*.

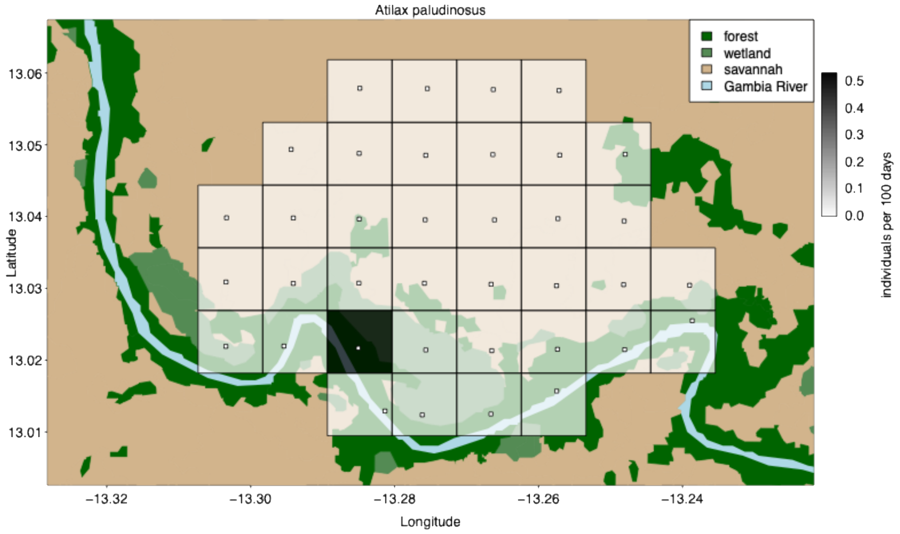

Figure S21: Number of individuals of *Atilax paludinosus*.

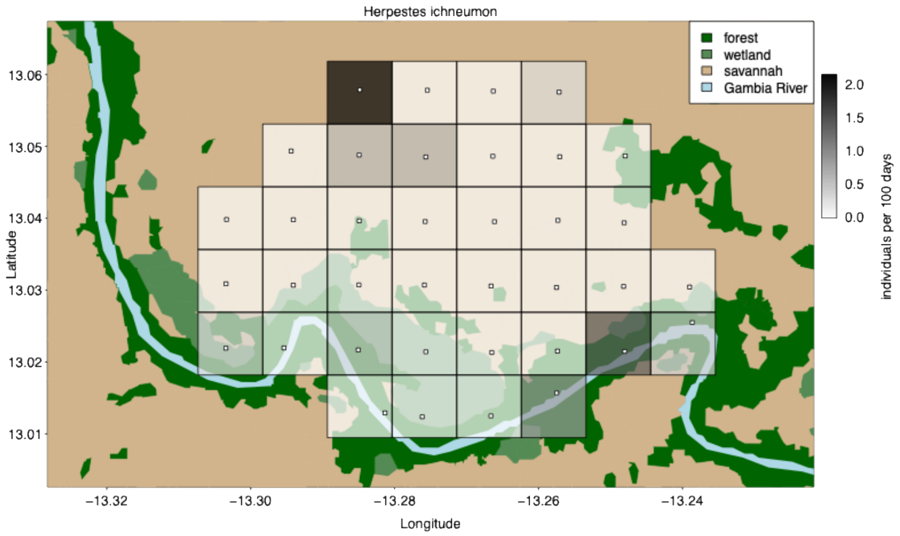

Figure S22: Number of individuals of *Herpestes ichneumon*.

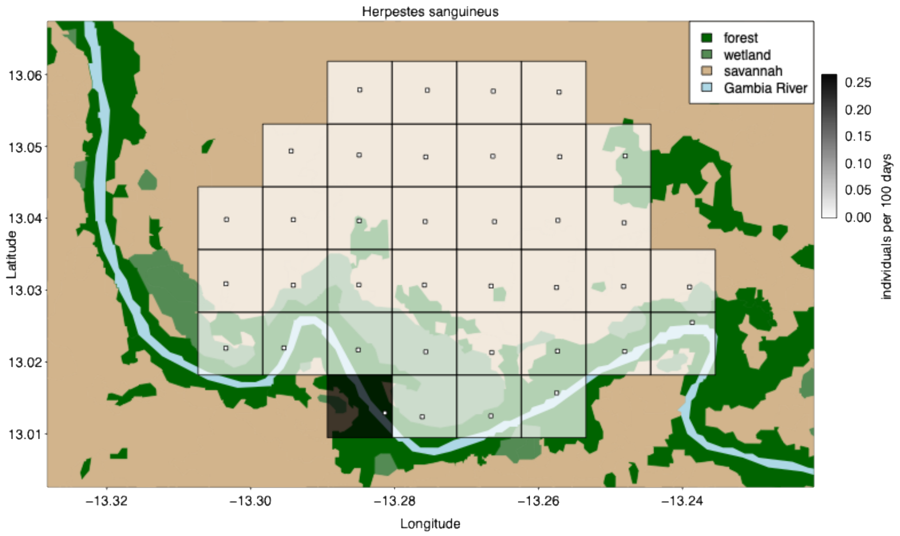

Figure S23: Number of individuals of *Herpestes sanguineus*.

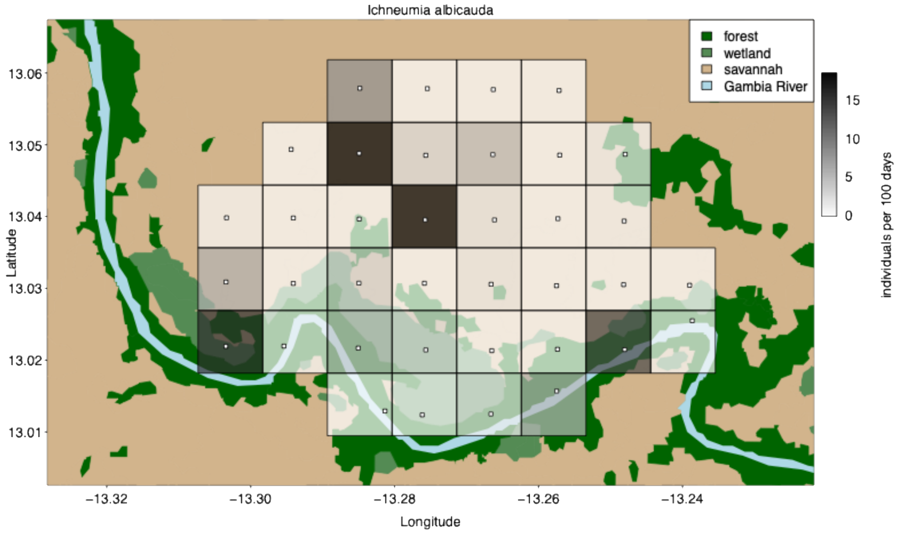

Figure S24: Number of individuals of *Ichneumia albicauda*.

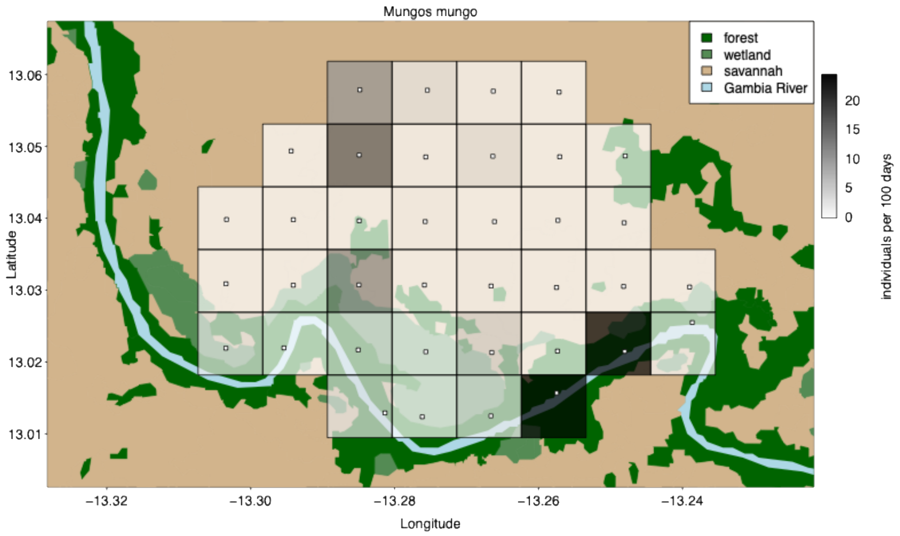

Figure S25: Number of individuals of *Mungos mungo*.

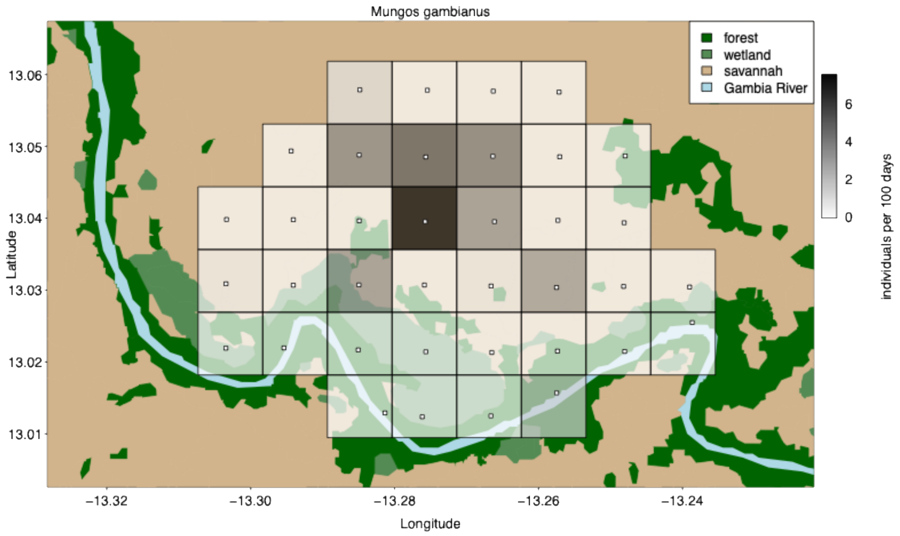

Figure S26: Number of individuals of *Mungos gambianus*.

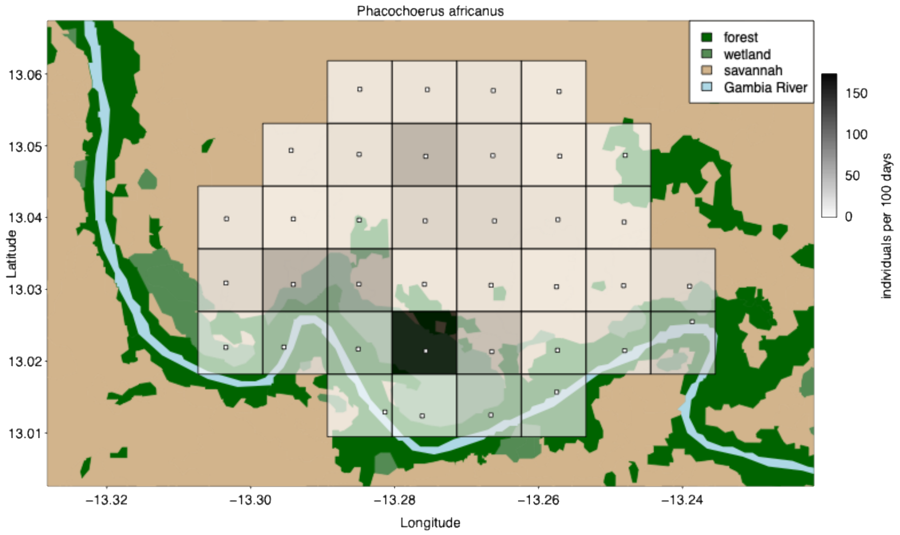

Figure S27: Number of individuals of *Phacochoerus africanus*.

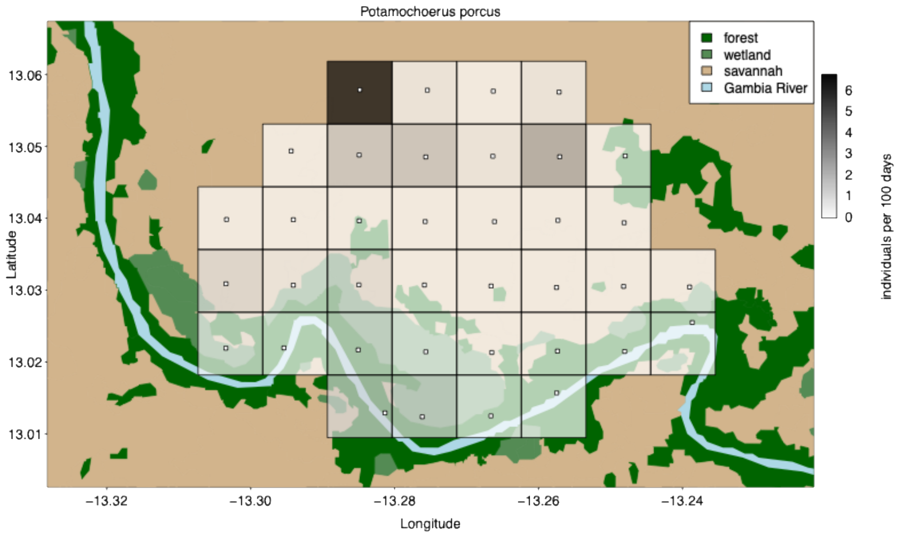

Figure S28: Number of individuals of *Potamochoerus porcus*.

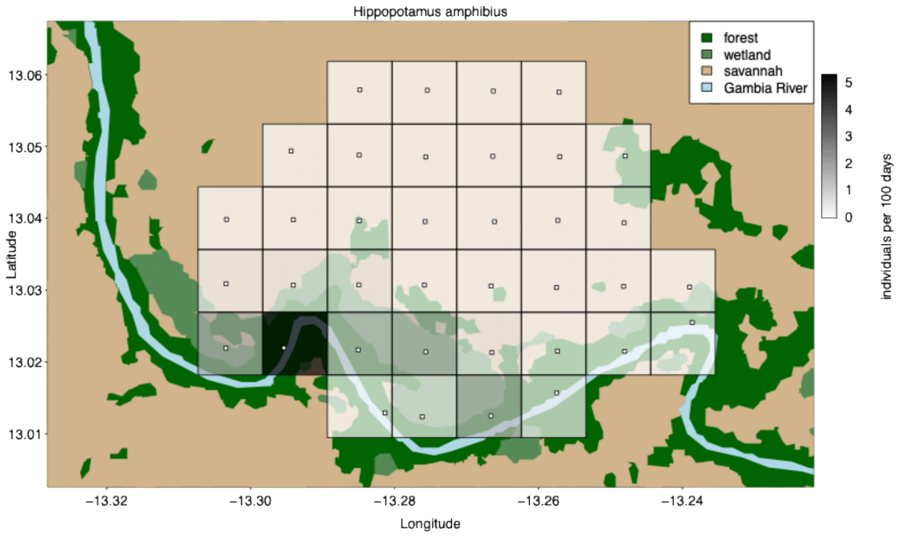

Figure S29: Number of individuals of *Hippopotamus amphibius*.

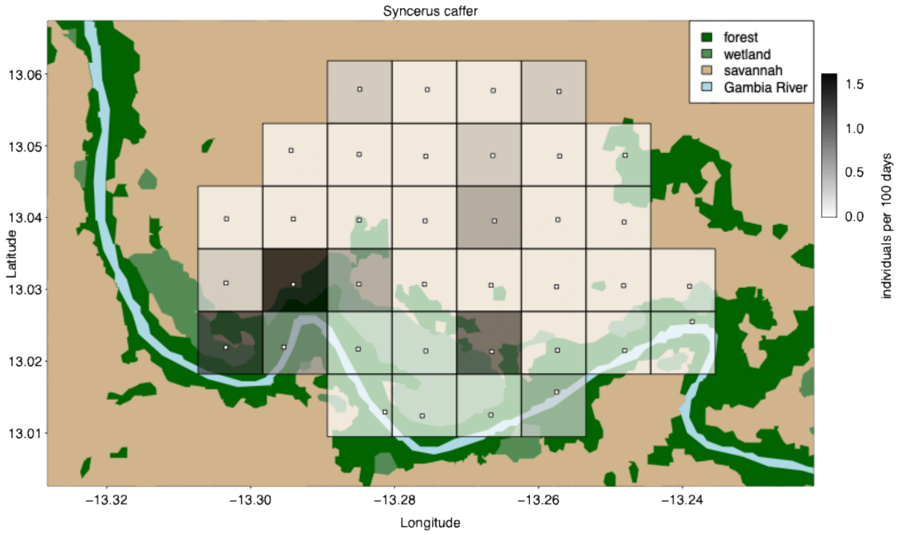

Figure S30: Number of individuals of *Syncerus caffer*.

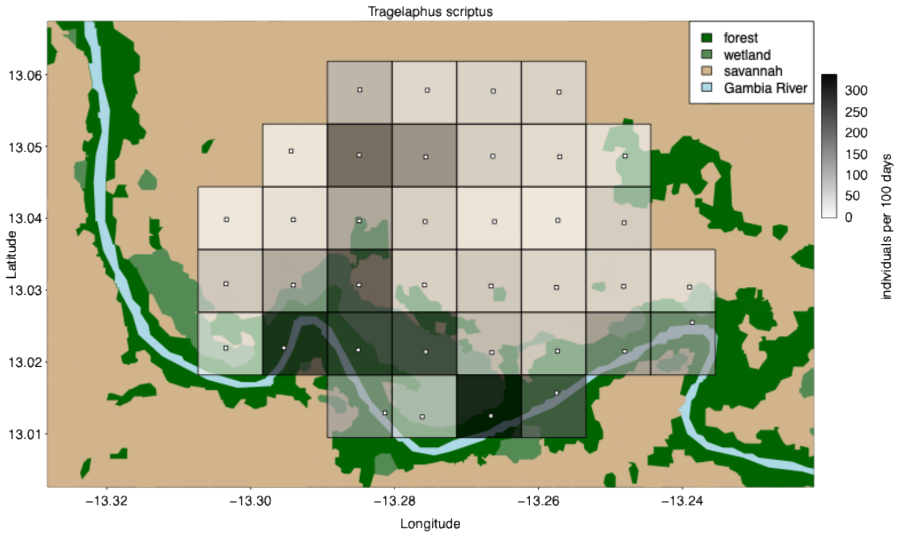

Figure S31: Number of individuals of *Tragelaphus scriptus*.

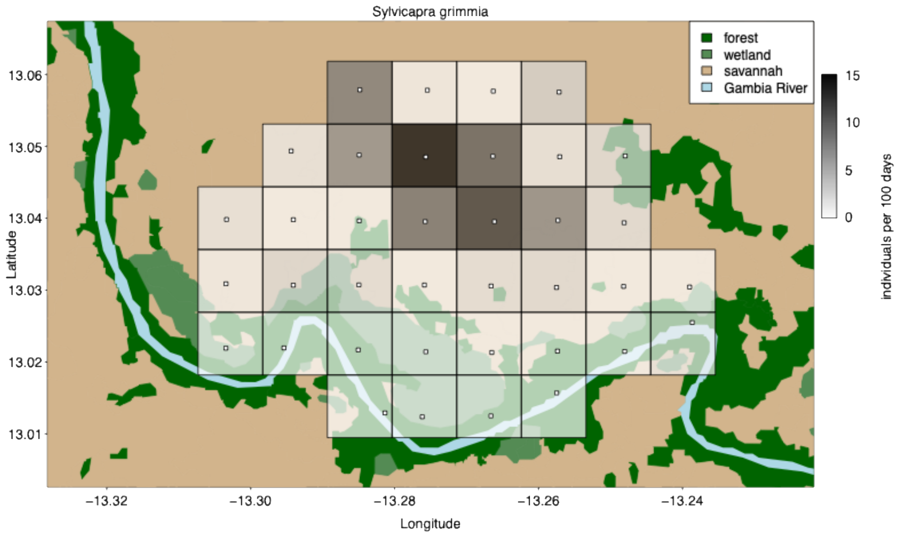

Figure S32: Number of individuals of *Sylvicapra grimmia*.

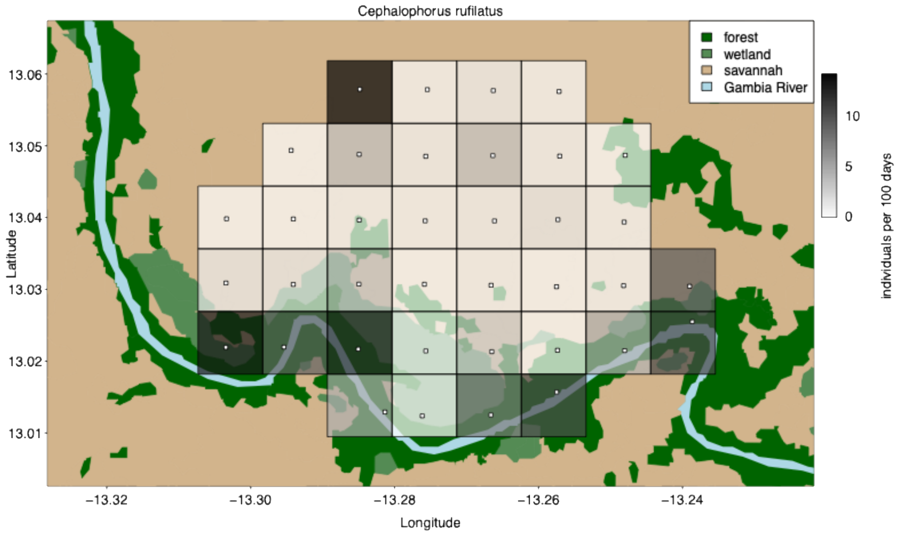

Figure S33: Number of individuals of *Cephalophorus rufilatus*.

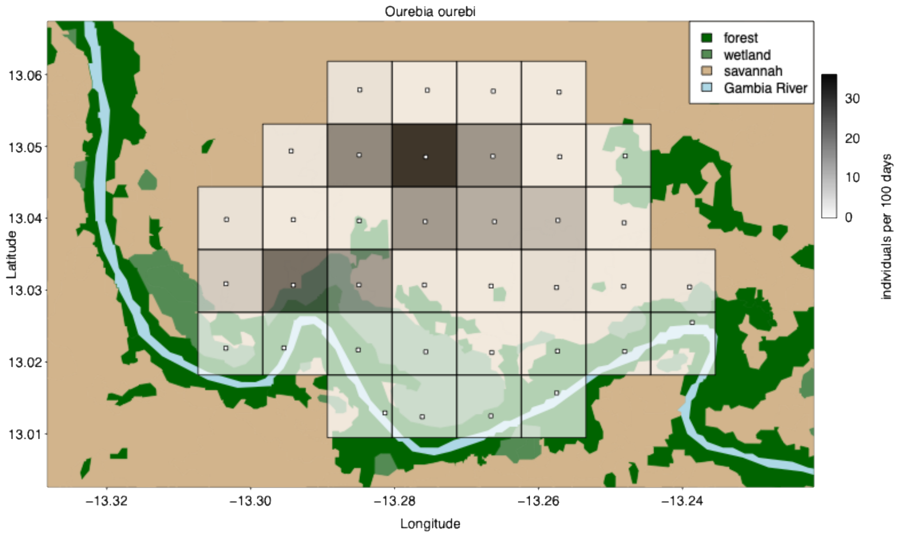

Figure S34: Number of individuals of *Ourebia ourebi*.

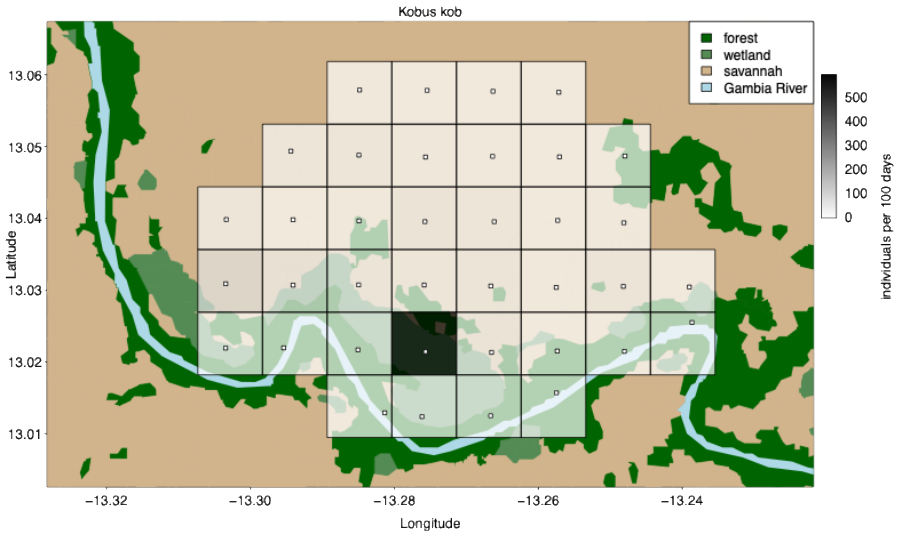

Figure S35: Number of individuals of *Kobus kob kob*.

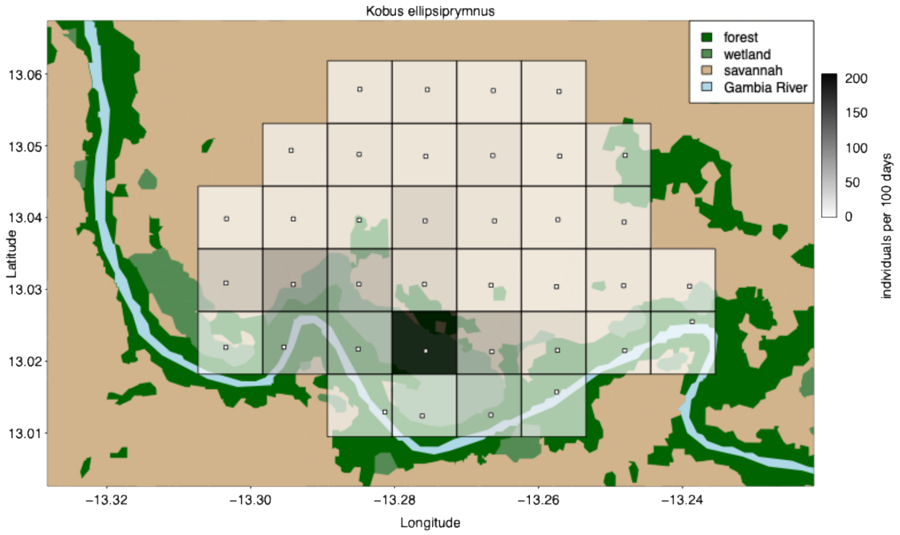

Figure S36: Number of individuals of *Kobus ellipsiprymnus defassa*.

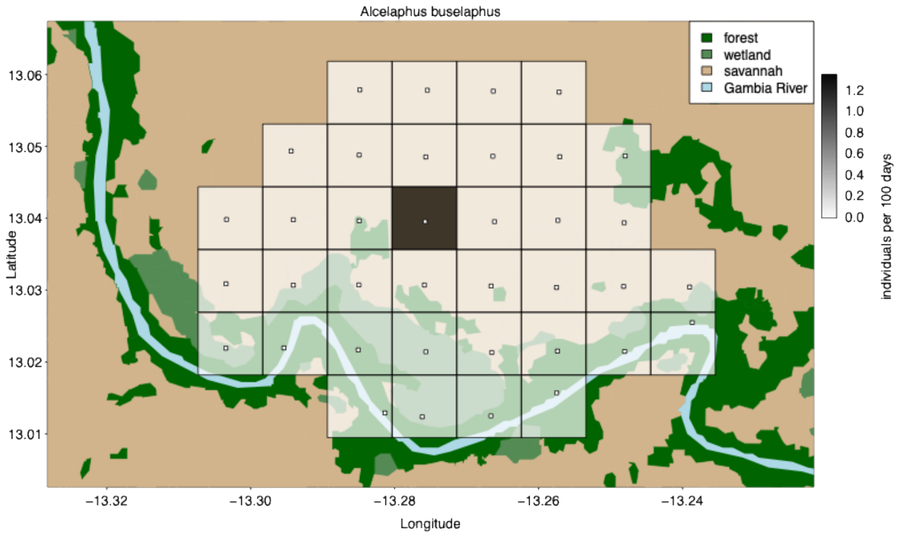

Figure S37: Number of individuals of *Alcelaphus buselaphus major*.

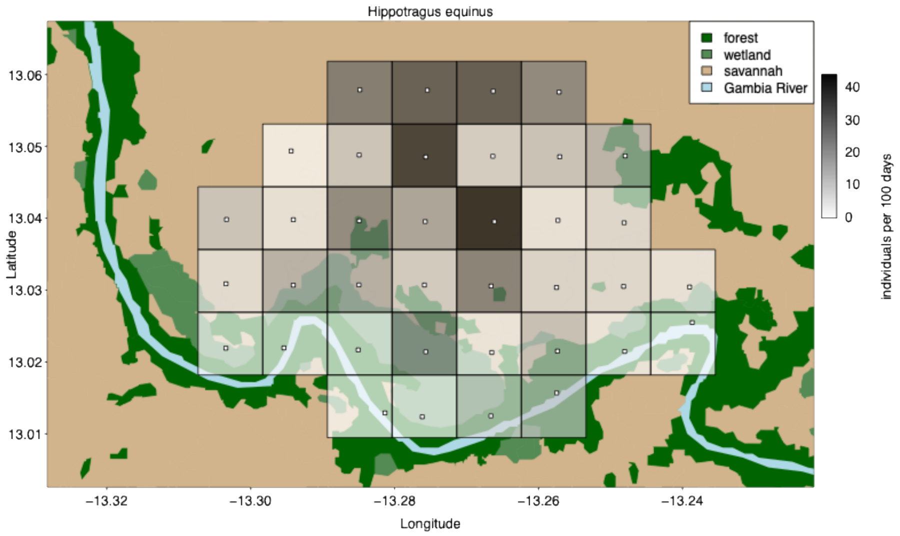

Figure S38: Number of individuals of *Hippotragus equinus koba*.
